# HAPRAP: a haplotype-based iterative method for statistical fine mapping using GWAS summary statistics

**DOI:** 10.1093/bioinformatics/btw565

**Published:** 2016-09-01

**Authors:** Jie Zheng, Santiago Rodriguez, Charles Laurin, Denis Baird, Lea Trela-Larsen, Mesut A Erzurumluoglu, Yi Zheng, Jon White, Claudia Giambartolomei, Delilah Zabaneh, Richard Morris, Meena Kumari, Juan P Casas, Aroon D Hingorani, UCLEB Consortium, David M Evans, Tom R Gaunt, Ian N M Day

**Affiliations:** 1 MRC Integrative Epidemiology Unit, School of Social and Community Medicine, Bristol, UK; 2 School of Social and Community Medicine, University of Bristol, Bristol, UK; 3 Department of Health Sciences, Genetic Epidemiology Group, University of Leicester, Leicester, UK; 4 Dedman College of Humanities and Sciences, Southern Methodist University, Dallas, TX, USA; 5 Department of Genetics, Environment and Evolution, University College London Genetics Institute, London, UK; 6 Department of Primary Care & Population Health, University College London, Royal Free Campus, London, UK; 7 Centre for Clinical Pharmacology, University College London, London, UK, Division of Medicine; 8 University of Queensland Diamantina Institute, Translational Research Institute, Brisbane, Australia, QLD

## Abstract

**Motivation:**

Fine mapping is a widely used approach for identifying the causal variant(s) at disease-associated loci. Standard methods (e.g. multiple regression) require individual level genotypes. Recent fine mapping methods using summary-level data require the pairwise correlation coefficients ($r^{2}$) of the variants. However, haplotypes rather than pairwise $r^{2}$, are the true biological representation of linkage disequilibrium (LD) among multiple loci. In this article, we present an empirical iterative method, HAPlotype Regional Association analysis Program (HAPRAP), that enables fine mapping using summary statistics and haplotype information from an individual-level reference panel.

**Results:**

Simulations with individual-level genotypes show that the results of HAPRAP and multiple regression are highly consistent. In simulation with summary-level data, we demonstrate that HAPRAP is less sensitive to poor LD estimates. In a parametric simulation using Genetic Investigation of ANthropometric Traits height data, HAPRAP performs well with a small training sample size (*N* < 2000) while other methods become suboptimal. Moreover, HAPRAP’s performance is not affected substantially by single nucleotide polymorphisms (SNPs) with low minor allele frequencies. We applied the method to existing quantitative trait and binary outcome meta-analyses (human height, QTc interval and gallbladder disease); all previous reported association signals were replicated and two additional variants were independently associated with human height. Due to the growing availability of summary level data, the value of HAPRAP is likely to increase markedly for future analyses (e.g. functional prediction and identification of instruments for Mendelian randomization).

**Availability and Implementation:**

The HAPRAP package and documentation are available at http://apps.biocompute.org.uk/haprap/

**Supplementary information:**

Supplementary data are available at *Bioinformatics* online.

## 1 Introduction

Genome-wide association studies (GWAS) have identified thousands of single nucleotide polymorphisms (SNPs) associated with human complex traits and diseases (Hindorff *et al.*, 2009; Manolio, 2010). To increase the power to detect small genetic effects associated with common complex traits, meta-analysis of multiple GWAS studies have also been conducted including blood lipids (Teslovich *et al.*, 2010, Electrocardiographic (ECG) traits (Arking *et al.*, 2006; Gaunt et al., 2012; Newton-Cheh et al., 2009; Pfeufer *et al.*, 2009) and human height (Wood *et al.*, 2014) amongst others. )

When a plausible hit has been identified within a GWAS, the challenge becomes one of determining the independent potentially causal SNP signals from a background of many correlated variants within the linkage disequilibrium (LD) block. A common strategy adopted is to take the top association signal to represent the association in a genomic region. However, this design does not take into account the possibility of multiple causal variants within a region, which will result in an underestimation of the total variation that could be explained at a locus (Yang *et al.*, 2012). Statistical methods are available to identify independent hits; however these methods either require access to individual level data, or rely on pairwise LD estimates when summary statistics are used.

Conditional analysis is time consuming when individual level genotype data from several cohorts needs to be analysed separately and then combined in meta-analysis (Zheng *et al.*, 2013). Providing the pairwise LD structure is consistent in samples from the same ethnic group (Ke *et al.*, 2004), there are two approximate conditional analysis methods that can effectively use GWAS summary data: Genome-wide Complex Trait Analysis (GCTA) conditional and joint effect analysis (COJO) (Yang *et al.*, 2012) and Sequential sentinel SNP Regional Association Plots (SSS-RAP) (Zheng *et al.*, 2013).

COJO is a state-of-the-art method extending the scope of multiple regression to summary-level meta-analysis. COJO estimates the approximate joint SNP effects from summary statistics in a meta-analysis and LD information from an appropriate reference sample. SSSRAP is a numerical and graphical approach that transforms the marginal SNP effect of a sentinel SNP to the joint SNP effect of a test SNP through a 2 × 2 SNP-haplotypes matrix.

These existing approximate conditional analysis methods use pairwise correlation coefficients (*r*^2^) between SNPs to represent LD structure in each associated region. However, when considering regions with three or more causal variants, utilizing allele frequencies and pair-wise LD correlation may lose LD information. Three-locus systems may place additional constraints on the maximum and minimum values for the pair-wise LD terms (Robinson *et al.*, 1991). Haplotypes, which represent combinations of co-inherited alleles within the same chromosome, are a more biologically correct way to represent LD among multiple loci. Fine mapping using haplotypes will pick up the LD information that is not detected using pairwise LD measures.

To aid the ‘missing LD information’ problem, we propose an empirical iterative method *HAPlotype Regional Association analysis Program* (HAPRAP) to improve the accuracy of approximate conditional analysis using GWAS summary data. The important difference between HAPRAP and COJO is that the former estimates the joint SNP effects by using haplotypes (rather than pair-wise LD) estimated from a reference sample. We use both simulations and real-data from the British Women’s Heart Health Study (BWHHS) (Lawlor *et al.*, 2003) to show that HAPRAP outperforms COJO on a range of performance measures. We applied the method to group-level QTc interval data from the UCL-LSHTM-Edinburgh-Bristol (UCLEB) meta-analysis (Shah *et al.*, 2013), with the haplotype information estimated from imputed genotype data from the BWHHS; and human height from the Genetic Investigation of ANthropometric Traits (GIANT) meta-analysis (Wood *et al.*, 2014), with the haplotype information estimated from the Avon Longitudinal Study of Parents and Children (ALSPAC). Both cases suggest that HAPRAP has increased power for fine mapping compared to COJO. We extended HAPRAP to binary phenotypes and we illustrate this with an example of meta-analysis for gallbladder disease (GBD) SNP hits (Rodriguez *et al.*, 2015).

## 2 Materials and methods

### 2.1 Overview of the methodology

We aim to combine summary level statistics with the full information from haplotypes (rather than using the traditional pairwise LD approach) to fine map genetic regions. Our algorithm iteratively updates haplotype effects based on haplotype frequencies and observed marginal SNP effects from meta-analyses to estimate the approximate joint SNP effect. This approach allows researchers to conduct conditional analysis more accurately without access to individual level genotypes.

#### 2.1.1 Theory

The haplotype-based approach we propose in this article is closely related to a single regression model. In a single regression model, we treat the major allele as the baseline allele; and the minor allele as the effect allele. The marginal SNP effect refers to the effect estimate from an outcome **Y** regressed on a single SNP (i.e. the allelic effect from a simple linear regression model). The joint SNP effect, which we aim to estimate, refers to the SNP effect obtained from Y regressed on multiple SNPs within the region. The joint SNP effect is adjusted for the correlation with surrounding SNPs, whereas the marginal SNP effect is not.

A simple extension of the single regression model to multi-locus data is to integrate two popular haplotype-based analysis strategies together: (i) dichotomize haplotypes into two groups (Lin and Zeng, 2006) and (ii) treat each group as a bivariate allele (Purcell *et al.*, 2007a).

Assume we obtain a SNP by haplotype matrix M, with $m_{k,j}=0\;or\;1$, from a sample population, we split existing haplotypes into two groups to estimate the joint effect of SNP *j*:

{HEj={l: ml,j=1}HBj={o: mo,j=0}

**Fig. 1:**
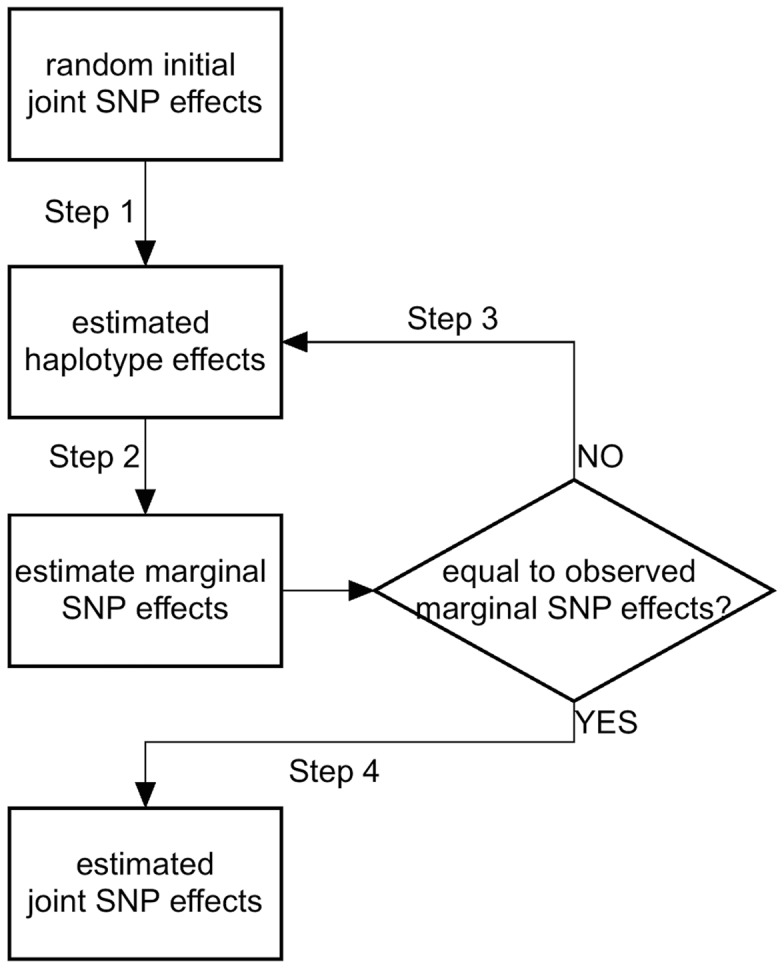
The SNP by haplotypes matrix for HAPRAP. The iteration of HAPRAP is built based on a matrix summarizing the haplotypes and haplotype frequencies for a certain population. ‘0’ in the matrix means the haplotype contains the baseline allele for the relevant SNP, whereas ‘1’ means the haplotype contains the effect allele for the relevant SNP. The small arrow (from left to right) is the marginal SNP effects estimation step. The large arrow (from right to left) is the haplotype effects adjustment step

${HE}_{j}$ is the set of haplotypes containing the effect allele of SNP *j*; and,$\;{HB}_{j}$ is the set of haplotypes containing the baseline allele of SNP *j*. For example for SNP1 in Figure 1, ${HE}_{1}$ is the set of haplotypes from Haplotypes 5–8, whereas ${HB}_{1}\;$is the set of haplotypes from Haplotypes 1–4. We also split the haplotype frequencies into two groups based on the relevant haplotypes $F_{l}$ and $F_{o}$.

We then define the estimated marginal SNP effect of a SNP *j*, $U_{j}$ as:

(1) $$U_{j}\;=\;z_{l,j}\;-\;z_{o,j};\;l\in {HE}_{j}\;and\;o\in {HB}_{j},\;$$

where $z_{l,j}$ (or $z_{o,j})\;$is the average of the additive effect over the set of haplotypes ${HE}_{j}\;\;(or\;{HB}_{j})$. These additive haplotype effects can be transferred to joint SNP effects using a generalized inverse matrix approach. This extension is applicable to both linear and logistic regression models.

#### 2.1.2 HAPRAP algorithm for estimating the joint SNP effect

**Fig. 2:**
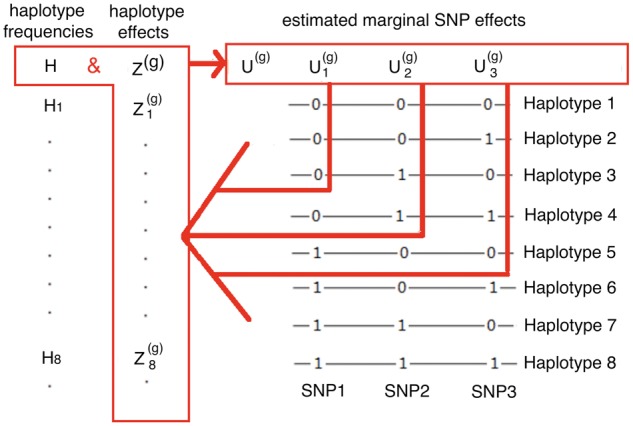
Schematic diagram of HAPRAP

As individual-level genotype data is usually not publicly available for GWAS meta-analysis, we cannot estimate haplotype effects by conducting a haplotype-based association analysis. Thus, we use an iterative method to estimate the haplotype effects from marginal SNP effects. The iteration involves four steps (Fig. 2):

Step 1: Setting initial values for joint SNP effects and haplotype effects transformation.
Step 2: The marginal SNP effects estimation.
Step 3: The haplotype effects adjustment.
Step 4: Convergence and the generalized inverse matrix approach.

Table 1 provides details of the notation used in describing our method.

**Table 1:** Notation of HAPRAP

Par.	Description
**M**	K×J SNP by haplotype matrix, with $m_{k,j}$ = 0 or 1, with 0 being the baseline allele of SNP *j*, 1 being the effect allele of SNP *j*
**HE**_*j*_	The set of haplotypes containing the effect allele of SNP *j*
**HB**_*j*_	The set of haplotypes containing the baseline allele of SNP *j*
**F**_*l*_	The set of haplotype frequencies containing the baseline allele of SNP *j*
**F**_*o*_	The set of haplotype frequencies containing the baseline allele of SNP *j*
**O**	J×1 vector of observed marginal SNP effects from GWAS/meta-analysis
**V**^(0)^	J×1 vector of random initial joint SNP effects
**U**^(g)^	J×1 vector of the estimated marginal SNP effects in the *g*th iteration
**Z**^(g)^	K×1 vector of the estimated haplotype effects of in the *g*th iteration
*x*^(g)^	The SNP with the greatest deviation between the observed marginal SNP effect and the estimated marginal SNP effect in the g iteration
**V**^(g)^	J×1 vector of the estimated joint SNP effects in the *g*th iteration

Column ‘Par.’ lists the parameters used in HAPRAP.

*Step 1. Setting initial values for joint SNP effects and haplotype effects transformation:* The algorithm starts with setting up a random set of initial joint effects for SNPs, $V^{\left(0\right)}$

Assuming that haplotypes (and haplotype frequencies) in the reference panel are the same as those in the GWAS meta-analysis, we estimate the haplotype frequencies F and the SNP by haplotype matrix M from the reference panel.

Assuming an additive linear model, the initial estimated haplotype effect $Z^{\left(0\right)}$ is the matrix product of M and $V^{\left(0\right)}$ (Fig. 1):

(2) $$MV^{\left(0\right)}=Z^{\left(0\right)}.\;$$

*Step 2. Marginal SNP effects estimation*: 

As mentioned in Equation (1), we define the marginal SNP effect as the difference between the sums of the additive effects of the two sets of haplotypes ${HE}_{j}\;and\;{HB}_{j}$.

Thus, for the g iteration, where g = {*0…G*}, the marginal SNP effect of SNP *j*, $U_{j}^{\left(g\right)}$, is estimated by counting the difference between the two groups of haplotype effects, $Z_{j,l}^{\left(g\right)}$ and $Z_{j,o}^{\left(g\right)}$, and standardized by the relevant haplotype frequencies, $F_{l}$ and $F_{o}$:

(3) $$U_{j}^{\left(g\right)}=\;\frac{1}{\sum_{l\in {HE}_{j}}F_{l}}\left(\sum_{l\in {HE}_{j}}F_{l}Z_{j,\;l}^{\left(g\right)}\right)-\;\frac{1}{\sum_{o\in {HB}_{j}}F_{o}}\left(\sum_{o\in {HB}_{j}}F_{o}Z_{j,\;o}^{\left(g\right)}\right).\;$$

We tested the reliability of Equation (3) by a simulation and found that given any set of joint SNP effects, application of Equation (3) never generated non-zero effect estimates for SNPs that were simulated to have truly null effects (Supplementary Text S2).

*Step 3. Haplotype effects adjustment*: the adjusted marginal SNP effects for iteration g, $U^{\left(g\right)}$ are compared to the observed marginal SNP effects, O. Reconciling the difference between $U^{\left(g\right)}$ and O is important because it equates the marginal SNP effects observed from the meta-analytic data with those that would arise under the distribution of haplotypes in the reference panel. The SNP with the greatest deviation, denoted $x^{\left(g\right)}$, is adjusted for the next iteration g + 1, the other SNP effects remain the same:

(4) Ujg+1= Ujg-Oi where j=x(g)Ujg where j ≠x(g). 

Then the haplotype effect $Z^{\left(g+1\right)}$ will be adjusted based on the change of $U_{j}^{\left(g+1\right)}$. For haplotype *k*, we get:

(5) $$Z_{k}^{\left(g+1\right)}=Z_{k}^{\left(g\right)}+U_{j}^{\left(g\right)}m_{k,j}\;where\;j=x^{\left(g\right)}.\;$$

*Step 4. Convergence and the generalized inverse matrix approach*: After the estimated marginal SNP effects, $U^{\left(g\right)}$ converge to within 10 decimal places of the observed SNP effects, O, we stop the iteration. The joint SNP effects, $V^{\left(g\right)}$, is estimated using the generalized inverse matrix approach:

(6) $$M^{-1}Z^{\left(g\right)}=V^{\left(g\right)}.\;$$

### 2.2 Estimating standard errors of the estimated joint SNP effects and testing SNP significance using parametric bootstrap

We estimate the standard errors (SE) of the estimated joint SNP effects using a bootstrap approach so that we can apply the stepwise elimination using the joint *P*-value in the next step.

#### 2.2.1 Pre-test of SNP significances

Generating bootstrap SE can use computational resources intensively. To improve computational efficiency, we first pre-test the significance of the candidate SNPs using the estimated joint SNP effects $V^{\left(g\right)}$ and the SE of the observed marginal SNP effects (since the uncertainty of the effect of a given SNP is larger in a multivariate model than that in a single SNP model). SNPs with the highest *P*-value will be step-wise eliminated from the model until all SNPs reach the *P*-value threshold we set.

If two or more SNPs remain in the model after the pre-test using SEs from single locus regression, we then estimate the SE of HAPRAP betas of these SNPs using a simulation based HAPRAP program (simHAPRAP) (Supplementary Fig. S1). The simHAPRAP program starts with simulating a population with sample size equal to the total number of participants in the meta-analysis. Genotypes for each individual are generated based on the haplotypes and haplotype frequencies. Quantitative phenotypes are simulated from a normal distribution with mean equal to zero and SE equal to the observed SD of the phenotype; whereas binary phenotypes are simulated from a binomial distribution which matches the observed probability of cases. A weighted genetic risk factor is used as the total genetic effect on the trait (Supplementary Fig. S1).

We repeat the simHAPRAP procedure 2000 times. The SE of the betas over the 2000 replications are used as the SE of the HAPRAP betas (defined here as simHR SE).

#### 2.2.2 Stepwise backwards elimination

*t*-test *P*-values are calculated using HAPRAP betas and simHAPRAP SEs. We backward eliminate the SNPs with the highest *P*-values until all SNPs in the model reach a pre-set *P*-value cut-off.

#### 2.2.3 HAPRAP availability

The HAPRAP software and a web-based instruction manual (developed using HTML and Cascading Style Sheets (CSS)) are available at http://apps.biocompute.org.uk/haprap.

### 2.3 Sample datasets

The real cases and simulated datasets we used for this analysis are explained in Supplementary Text S3.

### 2.4 Simulation framework and empirical comparison

Firstly, we simulated a pool of 100 000 individuals (details in Supplementary Text S3) and performed a series of simulations to test the influence of LD structure and sample size of reference panel. For each model explained in Supplementary Text S3 and Supplementary Table S1, we applied HAPRAP and COJO to the summary statistics and the genotypes of a specific reference panel. We also applied multiple regression using individual-level phenotypes and genotypes from the reference panel. For each method, the mean and SD of the joint SNP effect were estimated 1000 times. In addition, multiple regressions on the 100 000 individuals were conducted (Supplementary Text S3) and the resulting joint SNP effects were set as the gold standards. Mean square error (MSE) of the gold standard effect was used to measure the accuracy of each method.

Secondly, we performed a parametric simulation to test the influence of the sample size of a meta-analysis. The GIANT height meta-analysis data were used as the basis of this simulation (Wood *et al.*, 2014). We selected 20 nearest SNPs from the *ACAN* region. ALSPAC pre-phased haplotypes of 8263 unrelated children were used to build a genotype pool for 253 288 individuals. We randomly selected 100 000, 50 000, 10 000, 5000, 2500, 1750 and 1000 individuals from the pool, comparing the performance of HAPRAP and COJO using multiple regression as the gold standard. 1000 replications were processed to estimate the MSE and SD of the MSE.

Thirdly, as an empirical comparison between HAPRAP and COJO, we explored these methods using real data from the BWHHS and the 1000 Genomes project (1000 Genome Project Consortium, 2010). Details of the performance comparisons are explained in Supplementary Text S4.

### 2.5 Case study for quantitative traits: GIANT height

We firstly applied HAPRAP to two meta-analyses. Details of these two case studies are explained in Supplementary Text S5. We further applied HAPRAP to summary-level data from the GIANT height meta-analysis (sample size 253 288). The pre-phased haplotypes of 8263 unrelated children from ALSPAC were used as the reference panel. Three genomic regions with more than one robust independent association signal were selected (Wood *et al.*, 2014). All SNPs within these regions were selected (782 SNPs for *ACAN*, 1477 SNPs for *ADAMTS17* and 1936 SNPs for *PTCH1*).

## 3 Results

### 3.1 Simulation and empirical comparison

Firstly, we fixed the sample size of the meta-analysis (*N* = 100 000) and compared the performance of HAPRAP and COJO across different LD structures and different sample sizes of reference panel using a simulation data set (details in Supplementary Text S3). As shown in Supplementary Table S2, HAPRAP outperformed COJO under a variety of LD structures and was less sensitive to poor LD estimation.

In the two-SNP models with one causal SNP and one non-effect SNP, HAPRAP was slightly (up to 29%) more accurate than COJO across 16 models (Supplementary Fig. S2A and Supplementary Table S2A). Both methods performed well when the sample size of the reference panel was larger than 5000. When the sample size of the reference panel was limited to 500–1000, HAPRAP started to outperform COJO. On the other hand, considering the influence of LD structure, HAPRAP was up to 54% more accurate than COJO when LD between the two SNPs was extremely high ($r^{2}$ = 0.9).

**Fig. 3:**
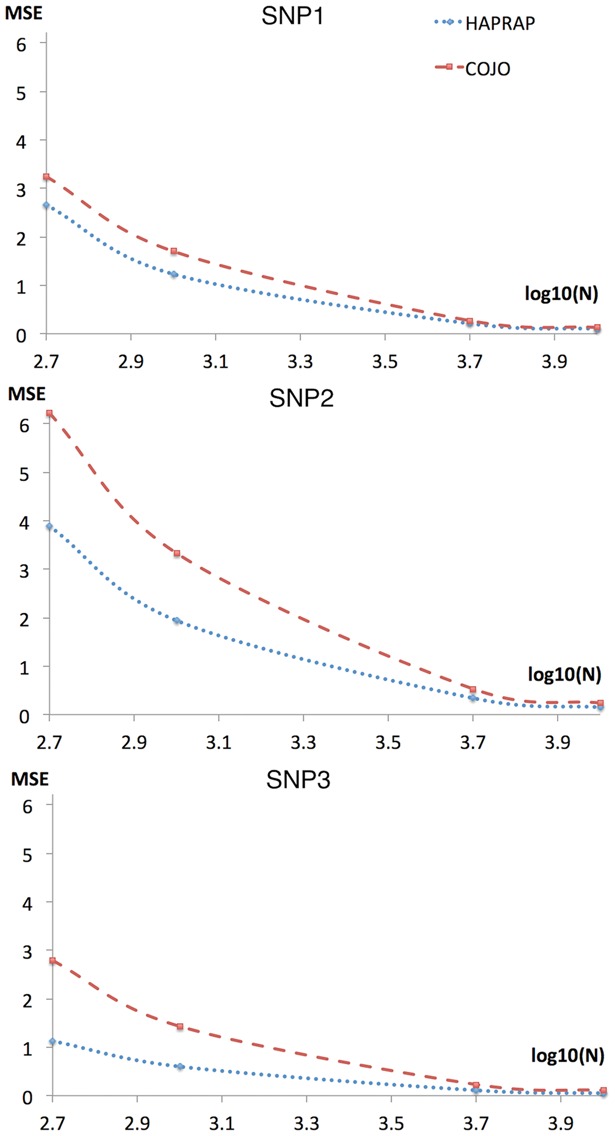
Performance comparison between HAPRAP and COJO in one of the three-SNPs Model MSE is MSE of HAPRAP (or that of GCTA) compare to joint effect from multiple regression mode. *X*-axis is the number of individuals in the reference panel on a log scale, which is equivalent to sample size of 10 000, 5000, 1000 or 500, respectively. In this simulation, SNP1 is a signal with a joint effect of 1, SNP2 is a bystander SNP with no effect, SNP3 is a secondary SNP with a joint effect of 0.3, $r^{2}$ between SNP1 and SNP2 was 0.8, $r^{2}$ between SNP1 and SNP3 was 0.5

In the three-SNP models with two causal SNPs and one non-effect SNP (Fig. 3 and Supplementary Fig. S2B), both methods performed relatively well when the sample size of the reference panel was larger than 5000 (although with more errors compared to the two-SNP models). However, both methods struggled to eliminate the non-effect SNP when the sample size of reference panel is <1000 and LD was very high amongst three SNPs. However, in a more realistic LD range ($r^{2}$ between each pair of SNPs from 0.1 to 0.5) and with a small reference sample size (*N* = 1000), HAPRAP was, on average, 63% more accurate than COJO (Supplementary Table S2B).

We demonstrated in this simulation that, when individual-level data is extremely limited, HAPRAP (using summary level data and a reference panel with a small number of individuals) is a better option than applying multiple regression to the reference panel with limited sample size (Supplementary Fig. S2C and Supplementary Table S2).

**Fig. 4:**
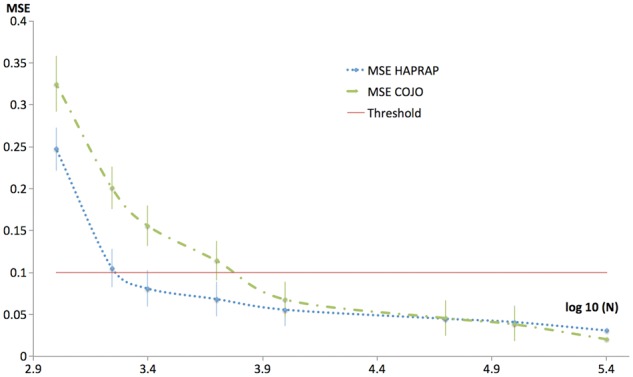
Performance comparison of HAPRAP and COJO using parametric simulation of 20 SNPs from GIANT height meta-analysis MSE is the MSE of the method compare to multiple regression. *X*-axis is the number of individuals in the meta-analysis in Log scale. Horizontal line is the threshold line of MSE of 0.1

Secondly, in the parametric simulation using GIANT height data, we assumed perfect LD estimation and only consider the influence of sample size of the meta-analysis. As shown in Figure 4 and Supplementary Table S3, HAPRAP and COJO were close to optimal (Supplementary Text S6 explains the reason COJO is not perfectly optimal in this situation) when the sample size of the meta-analysis was large (*N* ≥ 10 000). When the training sample size was between 1750 and 5000, HAPRAP’s MSE was still under 0.1 while COJO became suboptimal.

Thirdly, we utilized individual-level data of ∼2000 BWHHS individuals on a total of 115 SNPs to compare the accuracy of HAPRAP [haplotypes phased by both segmented haplotype estimation and imputation tool (SHAPEIT) (Delaneau *et al.*, 2012) and PLINK] and COJO using multiple regression as the gold standard (Supplementary Table S4). The details of the comparison can be found in Supplementary Text S4. In summary, the comparisons suggested that HAPRAP was comparable to multiple regression when the individual-level genotypes are available for the entire cohort. In addition, HAPRAP was on average 10.86% more accurate than COJO when the sample size of the reference panel was extremely limited (Sample size <200).

### 3.2 Case study: GIANT meta-analysis of height

We further analysed three genomic regions reported to be associated with human height by the GIANT consortium. The original fine mapping analyses were processed using COJO, resulting in 18 associated SNPs with *P*-value <5 × 10^−8^ at these 3 loci (Wood *et al.*, 2014). Here, we applied HAPRAP to a total of 4195 SNPs using 8263 unrelated ALSPAC children as a reference panel. The allele frequencies of GIANT and the ALSPAC children were quite similar (Supplementary Table S5). As shown in Table 2, HAPRAP replicated all 18 previously reported association signals at these 3 loci (Table 2). Moreover, HAPRAP identified two novel signals, rs1529889 (an intronic variant in *ADAMST17* with joint effect of 0.019) and rs357564 (a missense variant in *PTCH1* with joint effect of −0.034), independently associated with height, (Table 2). As shown in Supplementary Table S6, these two SNPs are in low LD with independent SNPs in the same genomic region.

**Table 2:** Summary of 20 associated SNPs at 3 loci for height with *P* < 5 × 10^−8^ in the HAPRAP step-wise model selection analysis using the ALSPAC cohort as a reference sample for LD

SNP	COJO-GIANT		HAPRAP	
	Beta	*P*-value	Beta	*P*-value
rs1348002	0.020	1.5 × 10^−10^	0.018	2.8 × 10^−09^
rs11633371	0.024	2.1 × 10^−15^	0.028	4.8 × 10^−20^
rs16942341	−0.114	3.0 × 10^−29^	−0.122	3.4 × 10^−34^
rs2280470	0.031	5.5 × 10^−21^	0.032	1.9 × 10^−25^
rs3817428	0.022	2.6 × 10^−09^	0.019	1.2 × 10^−08^
rs2238300	−0.018	1.6 × 10^−09^	−0.020	3.8 × 10^−11^
rs2573625	0.030	3.7 × 10^−22^	0.025	2.4 × 10^−15^
rs1529889	Unselected	Unselected	0.019	6.4 × 10^−10^
rs4246302	−0.027	1.4 × 10^−16^	−0.028	1.4 × 10^−17^
rs4548838	0.034	9.1 × 10^−30^	0.033	1.4 × 10^−28^
rs7170986	−0.019	1.1 × 10^−08^	−0.018	4.5 × 10^−08^
rs8042424	−0.022	5.1 × 10^−10^	−0.022	2.2 × 10^−10^
rs1257763	0.071	9.4 × 10^−14^	0.078	2.2 × 10^−12^
rs12347744	−0.056	2.8 × 10^−20^	−0.039	1.7 × 10^−19^
rs357564	Unselected	Unselected	−0.046	3.9 × 10^−13^
rs4448343	−0.035	1.1 × 10^−28^	−0.035	2.0 × 10^−17^
rs1329393	0.038	1.4 × 10^−15^	0.034	5.1 × 10^−13^
rs817300	−0.070	2.2 × 10^−23^	−0.085	4.8 × 10^−16^
rs10990303	0.032	1.4 × 10^−19^	0.036	5.4 × 10^−18^
rs7870753	−0.045	1.7 × 10^−37^	−0.043	1.3 × 10^−30^

Beta and *P*-value under COJO-GIANT refer to the joint SNP effect and its *P*-value presented in the GIANT height paper. Beta and *P*-value under HAPRAP are the joint SNP effect and its *P*-value for HAPRAP. ‘Unselected’ means the SNP was not selected by COJO in the step-wise selection. The comparison details are presented in Table S5.

Surprisingly, when we applied COJO to the same data using a different reference panel (ALSPAC instead of ARIC), only 16 SNPs were significantly associated with height, leaving two SNPs unselected (Supplementary Table S5).

We also conducted two case studies of GBD and QTc intervals. Details of these case studies are in Supplementary Text S5.

## 4 Discussion

Meta-analysis summary association statistics are becoming more and more widely available to the scientific community (Bulik-Sullivan *et al.*, 2015a). Several genetic analysis methods have been developed to exploit these resources (using summary rather than individual-level data), for example LD score regression (Bulik-Sullivan *et al.*, 2015a,b; Finucane *et al.*, 2015), Gaussian imputation (Pasaniuc *et al.*, 2014) and two-sample Mendelian randomization (Pierce and Burgess, 2013).

In this article, we introduced a novel approach for statistical fine mapping using meta-analysis summary statistics. The proposed method (HAPRAP) uses haplotypes to represent LD structure among multiple variants in a region. Using haplotypes has four significant advantages compared to existing conditional analysis methods that utilize pairwise correlation coefficients (*r*^2^) between SNPs [such as COJO (Yang *et al.*, 2012) and SSSRAP (Zheng *et al.*, 2013)]:

1. It considers all loci simultaneously, rather than pairwise, thus it is less susceptible to poor LD estimates that occur if the reference LD structure does not closely match the populations studied in the GWAS data.
2. It is more accurate than COJO when the sample size of the meta-analysis is limited (e.g. *N* ≤ 5000).
3. It is more accurate and powerful for regions with three or more independent signals. Compared to Bayesian fine mapping methods such as Probabilistic Annotation INtegraTOR (PAINTOR) (Kichaev *et al.*, 2014; Kichaev and Pasaniuc 2015), CAusal Variants Identification in Associated Regions (CAVIAR) (Hormozdiari *et al.*, 2014) and CAVIAR Bayes factor (CAVIARBF) (Chen *et al.*, 2015), HAPRAP does not require the user to specify the number of causal variants. This can impair the performance of CAVIARBF for cases where there are multiple causal variants (Kichaev *et al.*, 2014). We observed a power improvement in our case study of human height (e.g. with 3+ independent signals within each associated region).
4. It is more accurate when analysing rare variants (i.e. minor allele frequency (MAF) < 0.01) than other methods using pair-wise LD.

Our empirical demonstration using the 1000 Genomes Project (1000 Genome Project Consortium, 2010) data comparison is meaningful in three aspects: Firstly, high quality haplotypes data, which is used by HAPRAP, are now widely available and should have already been pre-phased within large-scale consortiums/cohorts such as the aforementioned 1000 Genomes Project and ALSPAC. Secondly, for researchers without individual-level genotype data, our method can give researchers a general profile of the potentially multiple associated SNPs in the region(s) of interest using the public available 1000 Genome Project data, although the errors of using the 1000 Genomes Project data as a reference panel were relatively large since the sample size is currently small. As more open access phased haplotype data becomes available with the publication of projects, such as UK10K (UK10K consortium, 2015), HAPRAP’s accuracy advantage against COJO will increase. Thirdly, HAPRAP’s performance advantage will be more apparent for GWAS studies with relatively smaller sample sizes, such as association analyses of DNA methylation with expensive or high-dimensional phenotypes [e.g. gene expression and methylation data (Gaunt *et al.*, 2015; Shi *et al.*, 2014)].

In the case study using summary statistics of GIANT data (Wood *et al.*, 2014), we identified two additional variants, rs1529889 and rs357564, independently associated with human height. These findings could have been caused by the greater sample size of the reference panel using ALSPAC (8263) compared to ARIC (6654). rs357564 is a missense variant within *PTCH1* and rs1529889 is an intronic variant within *ADAMST17*. rs357564 is predicted to be ‘functional’ by the prediction tool FATHMM (Shihab *et al.*, 2015) and was reported to be associated with oral clefts, basal cell carcinoma and ameloblastoma (Begnini *et al.*, 2010; Carter *et al.*, 2010; Farias *et al.*, 2012).

Rare variants are on average younger than common variants (Mathieson and McVean, 2014) and are more likely to be represented by longer haplotypes. Since HAPRAP uses haplotypes and COJO uses pairwise LD, we show HAPRAP may have a theoretical advantage over COJO in rare variant analyses. We performed a simulation for two SNPs with MAFs near 0.08 (Supplementary Table S7) and HAPRAP’s accuracy was higher than COJO in all conditions. Moreover, we highlighted a rare variant in *Apolipoprotein B* (*APOB*), rs41288783, as a proof-of-concept using real data (Supplementary Table S8). This SNP had a MAF of 0.0018 in BWHHS individuals. The HAPRAP estimate (beta = 0.705) is very close to the gold standard results (beta = 0.731), whereas the COJO estimate is considerably different from the gold standard (beta = 0.449).

We recommend using pre-phased haplotypes as HAPRAP input. For a cohort without haplotype data, we recommend users phase haplotypes using tools such as SHAPEIT (Delaneau *et al.*, 2012), BEAGLE (Browning and Browning, 2009), IMPUTE2 (Howie *et al.*, 2009) and Markov Chain Haplotyping algorithm (MACH) (Li *et al.*, 2010) rather than PLINK (Purcell *et al.*, 2007b). PLINK haplotype phasing function uses an E-M algorithm, which is only accurate and fast when a small number of SNPs (*N* < 10) are included (Browning and Browning, 2011).

We also suggest controlling for collinearity before utilizing HAPRAP. If SNPs with very high variance inflation factor (VIF) values are included, HAPRAP (and other tools) will return extremely large betas for a pair of SNPs. Practically, it is necessary to remove SNPs with VIF higher than seven before applying HAPRAP.

HAPRAP requires more time than COJO to finalize the step-wise elimination process. There are several reasons: firstly, phasing haplotypes is time consuming; secondly, it is time consuming to determine the SE of the joint SNP effects using our bootstrap method (simHAPRAP). However, the whole process does not usually take more than an hour.

HAPRAP was originally designed for regional fine mapping, so it is more suitable for moderately small numbers of markers and computationally very fast when the number of SNPs in each test is 10 or fewer. To fit the HAPRAP framework to fine map the whole genome, we recommend splitting regions with large numbers of SNPs into smaller chunks (up to 20 SNPs in each chunk) before running HAPRAP. In the GIANT height example, we split the genomic regions based on recombination hotspots, since LD patterns are directly related to the underlying recombination process, which is a more reasonable option compared to the physical distance used by COJO. This can help reduce the run time of HAPRAP substantially.

Algorithms are often used effectively where the biological model is well understood, but the statistical model is too complex to generalize to all scenarios. For instance, a recent fine mapping method, probability identification of causal SNPs (PICS), used an empirical constant in its core algorithm to estimate the expected mean of the association signal at a SNP (Farh *et al.*, 2015). HAPRAP interprets a complex biological concept, haplotype effects, using a simple idea stemming from allelic association analyses and extending it to the haplotype model. The side effect is that an asymptotic analysis of convergence may not be possible, thus we cannot exclude the possibility that HAPRAP will not converge in some situations. However, in the hundreds of thousands of simulations and real case examples we have tested, we did not find any situation where HAPRAP did not converge.

In a recent review paper (Spain and Barrett, 2015), fine mapping methods were classified into two groups: (i) methods for triaging variants based on *P*-values or LD with the lead SNP, which includes classic conditional analysis and approximate methods such as COJO and HAPRAP; (ii) Bayesian methods that assign posterior probabilities of membership in causal models to each SNP, such as PAINTOR, CAVIAR, CAVIARBF and the most recent software, FINEMAP (Benner *et al.*, 2016). Compared to CAVIARBF, FINEMAP used a new search algorithm and so is much faster and overcomes the limitation of situations where there are more than three causal variants in a genomic region. In addition, for the above Bayesian methods (with the exception of FINEMAP), a parameter must be set for the number of causal SNPs (Spain and Barrett, 2015). It has been shown that specifying this value to one can impair performance in cases where there are two or more causal variants (Kichaev *et al.*, 2014). Based on this we consider HAPRAP and these Bayesian methods as complementary. It would be interesting to explore the potential of integrating the HAPRAP methods with these Bayesian algorithms to develop more powerful fine mapping methods in the future.

In conclusion, with increasing numbers of publicly available meta-analysis summary statistics, the value of HAPRAP is likely to be demonstrated in four ways: (i) for fine mapping both common and rare variants and identifying additional variants independently associated with complex traits; (ii) it can be used as a variable selection method for two-sample Mendelian randomization; (iii) to build genome-wide allelic scores of biological intermediates for mining the phenome (Evans *et al.*, 2013); (iv) to provide a solid platform for the functional annotation of casual variants using prediction tools such as FATHMM (Supplementary Text S7).

## Supplementary Material

Supplementary Data
