# Supplementary material for: HAPRAP: a haplotype-based iterative method for statistical fine mapping using GWAS summary statistics
Source: Bioinformatics. 2016 Sep 1;33(1):79–86. doi: 10.1093/bioinformatics/btw565 (PMC5544112; doi:10.1093/bioinformatics/btw565)
Supplement: Supplementary Data [file btw565_supp.zip › btw565_Supp.docx]

**Table S1.** Assumptions of Pair-Wise Linkage Disequilibrium (r^2^) of SNPs Models for Population Simulations.

**A**

| 2 SNPs Model | |
| --- | --- |
| model | Rsq_S1&S2 |
| 1 | 0.9 |
| 2 | 0.8 |
| 3 | 0.5 |
| 4 | 0.2 |

**B**

| 3 SNPs Model | | |
| --- | --- | --- |
| model | Rsq_S1&S2 | Rsq_S1&S3 |
| 1 | 0.8 | 0.5 |
| 2 | 0.8 | 0.3 |
| 3 | 0.8 | 0.1 |
| 4 | 0.5 | 0.5 |
| 5 | 0.5 | 0.3 |
| 6 | 0.5 | 0.1 |

**A,** four 2-SNPs models with different pair-wise r^2^ between SNP1 and SNP2. **B,** six 3-SNPs models with different pair-wise r^2^ between SNP1 & SNP2 and SNP1 & SNP3.

**Table S2.** Performance Comparison of HAPRAP, GCTA and Multiple Regression using Artificial Meta-Analyses of the Simulated Populations.

**A**

| **2 SNPs model, Rsquare between SNP1 and SNP2 are showed below** | | | | | | | | | | | | | | |
| --- | --- | --- | --- | --- | --- | --- | --- | --- | --- | --- | --- | --- | --- | --- |
| SNP1 | | | | | | |  | SNP2 | | | | | | |
| Rsquare | N | Mean Square Error | | | comparison | |  | Rsquare | N | Mean Square Error | | | comparison | |
|  |  | HR | GCTA | Mreg | GCTA/HR | Mreg/HR |  |  |  | HR | GCTA | Mreg | GCTA/HR | Mreg/HR |
| Rsq=0.2 | 10K | 0.02 | 0.02 | 2.73 | 1.00 | 136.50 |  | Rsq=0.2 | 10K | 0.09 | 0.11 | 2.72 | 1.22 | 30.22 |
|  | 5K | 0.03 | 0.04 | 5.93 | 1.33 | 197.67 |  |  | 5K | 0.18 | 0.22 | 5.97 | 1.22 | 33.17 |
|  | 1K | 0.2 | 0.22 | 30.4 | 1.10 | 152.00 |  |  | 1K | 1.06 | 1.2 | 32.2 | 1.13 | 30.38 |
|  | 500 | 0.35 | 0.41 | 69.12 | 1.17 | 197.49 |  |  | 500 | 1.8 | 2.15 | 67.92 | 1.19 | 37.73 |
| Rsq=0.5 | 10K | 0.06 | 0.08 | 4.42 | 1.33 | 73.67 |  | Rsq=0.5 | 10K | 0.12 | 0.16 | 4.81 | 1.33 | 40.08 |
|  | 5K | 0.13 | 0.15 | 9.69 | 1.15 | 74.54 |  |  | 5K | 0.25 | 0.3 | 10.59 | 1.20 | 42.36 |
|  | 1K | 0.66 | 0.82 | 48.81 | 1.24 | 73.95 |  |  | 1K | 1.29 | 1.61 | 51.8 | 1.25 | 40.16 |
|  | 500 | 1.47 | 1.83 | 102.09 | 1.24 | 69.45 |  |  | 500 | 2.88 | 3.59 | 108.95 | 1.25 | 37.83 |
| Rsq=0.8 | 10K | 0.18 | 0.22 | 11.15 | 1.22 | 61.94 |  | Rsq=0.8 | 10K | 0.23 | 0.28 | 11.47 | 1.22 | 49.87 |
|  | 5K | 0.38 | 0.47 | 23.97 | 1.24 | 63.08 |  |  | 5K | 0.48 | 0.6 | 23.74 | 1.25 | 49.46 |
|  | 1K | 2.13 | 2.67 | 128.95 | 1.25 | 60.54 |  |  | 1K | 2.69 | 3.39 | 132.26 | 1.26 | 49.17 |
|  | 500 | 4.49 | 5.42 | 254.07 | 1.21 | 56.59 |  |  | 500 | 5.61 | 6.83 | 250.06 | 1.22 | 44.57 |
| Rsq=0.9 | 10K | 0.31 | 0.45 | 23.77 | 1.45 | 76.68 |  | Rsq=0.9 | 10K | 0.35 | 0.52 | 24.07 | 1.49 | 68.77 |
|  | 5K | 0.71 | 1 | 50.16 | 1.41 | 70.65 |  |  | 5K | 0.79 | 1.13 | 52.43 | 1.43 | 66.37 |
|  | 1K | 3.68 | 5.61 | 248.65 | 1.52 | 67.57 |  |  | 1K | 4.11 | 6.31 | 260.85 | 1.54 | 63.47 |
|  | 500 | 9.6 | 13.38 | 527.25 | 1.39 | 54.92 |  |  | 500 | 10.62 | 14.96 | 534.46 | 1.41 | 50.33 |
| Total | | 24.39 | 32.8 | 1541.16 | 20.28 | 1487.23 |  | Total | | 32.57 | 43.35 | 1574.3 | 20.60 | 733.93 |
| Average | | 1.52 | 2.05 | 96.32 | 1.27 | 92.95 |  | Average | | 2.04 | 2.71 | 98.39 | 1.29 | 45.87 |

**B**

| **3 SNPs model, Rsquare between SNP1 and SNP2 is 0.8, Rsquare between SNP1 and SNP3 are showed below** | | | | | | | | | | | | | | | | | | | | | | |
| --- | --- | --- | --- | --- | --- | --- | --- | --- | --- | --- | --- | --- | --- | --- | --- | --- | --- | --- | --- | --- | --- | --- |
| SNP1 | | | | | | |  | SNP2 | | | | | | |  | SNP3 | | | | | | |
| Rsquare | N | Mean Square Error | | | comparison | |  | Rsquare | N | Mean Square Error | | | comparison | |  | Rsquare | N | Mean Square Error | | | comparison | |
|  |  | HR | GCTA | Mreg | GCTA/HR | Mreg/HR |  |  |  | HR | GCTA | Mreg | GCTA/HR | Mreg/HR |  |  |  | HR | GCTA | Mreg | GCTA/HR | Mreg/HR |
| Rsq=0.1 | 10K | 0.09 | 0.13 | 11.35 | 1.44 | 126.11 |  | Rsq=0.1 | 10K | 0.13 | 0.19 | 11.79 | 1.46 | 90.69 |  | Rsq=0.1 | 10K | 0.08 | 0.1 | 3.15 | 1.25 | 39.38 |
|  | 5K | 0.21 | 0.31 | 23.1 | 1.48 | 110.00 |  |  | 5K | 0.32 | 0.44 | 25.97 | 1.38 | 81.16 |  |  | 5K | 0.19 | 0.23 | 6.58 | 1.21 | 34.63 |
|  | 1k | 1.06 | 1.47 | 117.61 | 1.39 | 110.95 |  |  | 1k | 1.55 | 2.13 | 130.47 | 1.37 | 84.17 |  |  | 1k | 0.93 | 1.17 | 33.51 | 1.26 | 36.03 |
|  | 500 | 2.31 | 3.42 | 256.63 | 1.48 | 111.10 |  |  | 500 | 3.36 | 4.95 | 284.41 | 1.47 | 84.65 |  |  | 500 | 2 | 2.55 | 71.46 | 1.28 | 35.73 |
|  | 200 | 6.69 | 10.11 | 665.49 | 1.51 | 99.48 |  |  | 200 | 9.24 | 13.99 | 727.21 | 1.51 | 78.70 |  |  | 200 | 5.14 | 6.44 | 175.71 | 1.25 | 34.18 |
|  | 175 | 7.59 | 11.41 | 736.52 | 1.50 | 97.04 |  |  | 175 | 10.75 | 16.03 | 834.16 | 1.49 | 77.60 |  |  | 175 | 5.6 | 7.11 | 206.66 | 1.27 | 36.90 |
|  | 150 | 14.5 | 21.35 | 946.36 | 1.47 | 65.27 |  |  | 150 | 18.63 | 27.41 | 1022.95 | 1.47 | 54.91 |  |  | 150 | 6.67 | 8.42 | 261.93 | 1.26 | 39.27 |
| Rsq=0.3 | 10K | 0.11 | 0.16 | 12.08 | 1.45 | 109.82 |  | Rsq=0.3 | 10K | 0.18 | 0.26 | 13.83 | 1.44 | 76.83 |  | Rsq=0.3 | 10K | 0.08 | 0.11 | 4.34 | 1.38 | 54.25 |
|  | 5K | 0.27 | 0.36 | 24.43 | 1.33 | 90.48 |  |  | 5K | 0.43 | 0.59 | 27.12 | 1.37 | 63.07 |  |  | 5K | 0.18 | 0.25 | 8.55 | 1.39 | 47.50 |
|  | 1k | 1.45 | 1.94 | 115.58 | 1.34 | 79.71 |  |  | 1k | 2.19 | 3 | 140.17 | 1.37 | 64.00 |  |  | 1k | 0.85 | 1.2 | 50.44 | 1.41 | 59.34 |
|  | 500 | 2.83 | 3.86 | 254.72 | 1.36 | 90.01 |  |  | 500 | 4.49 | 6.2 | 311.7 | 1.38 | 69.42 |  |  | 500 | 1.91 | 2.79 | 97.94 | 1.46 | 51.28 |
|  | 200 | 8.92 | 13.12 | 653.86 | 1.47 | 73.30 |  |  | 200 | 12.66 | 19.19 | 802.86 | 1.52 | 63.42 |  |  | 200 | 4.7 | 6.84 | 251.03 | 1.46 | 53.41 |
|  | 175 | 9.79 | 13.55 | 734.17 | 1.38 | 74.99 |  |  | 175 | 14.37 | 20.57 | 893.05 | 1.43 | 62.15 |  |  | 175 | 5.43 | 8.16 | 265.95 | 1.50 | 48.98 |
|  | 150 | 14.43 | 17.89 | 961.19 | 1.24 | 66.61 |  |  | 150 | 23.9 | 25.52 | 1110.31 | 1.07 | 46.46 |  |  | 150 | 6.18 | 8.88 | 338.44 | 1.44 | 54.76 |
| Rsq=0.5 | 10K | 0.1 | 0.13 | 11.83 | 1.30 | 118.30 |  | Rsq=0.5 | 10K | 0.15 | 0.24 | 17.69 | 1.60 | 117.93 |  | Rsq=0.5 | 10K | 0.05 | 0.11 | 7.33 | 2.20 | 146.60 |
|  | 5K | 0.21 | 0.27 | 25.02 | 1.29 | 119.14 |  |  | 5K | 0.34 | 0.53 | 35.31 | 1.56 | 103.85 |  |  | 5K | 0.11 | 0.23 | 15.5 | 2.09 | 140.91 |
|  | 1k | 1.23 | 1.7 | 142.97 | 1.38 | 116.24 |  |  | 1k | 1.94 | 3.33 | 194.16 | 1.72 | 100.08 |  |  | 1k | 0.6 | 1.43 | 81.43 | 2.38 | 135.72 |
|  | 500 | 2.66 | 3.25 | 296.65 | 1.22 | 111.52 |  |  | 500 | 3.9 | 6.22 | 406.6 | 1.59 | 104.26 |  |  | 500 | 1.13 | 2.78 | 163.84 | 2.46 | 144.99 |
|  | 200 | 10.81 | 15.1 | 644.52 | 1.40 | 59.62 |  |  | 200 | 14.57 | 23.9 | 919.16 | 1.64 | 63.09 |  |  | 200 | 3.1 | 7.52 | 428.14 | 2.43 | 138.11 |
|  | 175 | 14.13 | 19.41 | 817.14 | 1.37 | 57.83 |  |  | 175 | 18.62 | 31.19 | 1131.52 | 1.68 | 60.77 |  |  | 175 | 3.75 | 9.78 | 505.92 | 2.61 | 134.91 |
|  | 150 | 16.57 | 22.64 | 954.19 | 1.37 | 57.59 |  |  | 150 | 21.53 | 35.12 | 1364.77 | 1.63 | 63.39 |  |  | 150 | 3.71 | 9.5 | 609.17 | 2.56 | 164.20 |
| Total | | 115.97 | 161.57 | 8405.43 | 29.19 | 1945.10 |  | Total | | 163.24 | 241.02 | 10405.22 | 31.16 | 1610.59 |  | Total | | 52.38 | 85.61 | 3587.01 | 35.54 | 1631.08 |
| Average | | 5.52 | 7.69 | 400.26 | 1.39 | 92.62 |  | Average | | 7.77 | 11.48 | 495.49 | 1.48 | 76.69 |  | Average | | 2.49 | 4.08 | 170.81 | 1.69 | 77.67 |
|  |  |  |  |  |  |  |  |  |  |  |  |  |  |  |  |  |  |  |  |  |  |  |
| **3 SNPs model, Rsquare between SNP1 and SNP2 is 0.5, Rsquare between SNP1 and SNP3 are showed below** | | | | | | | | | | | | | | | | | | | | | | |
| SNP1 | | | | | | |  | SNP2 | | | | | | |  | SNP3 | | | | | | |
| Rsquare | N | Mean Square Error | | | comparison | |  | Rsquare | N | Mean Square Error | | | comparison | |  | Rsquare | N | Mean Square Error | | | comparison | |
|  |  | HR | GCTA | Mreg | GCTA/HR | Mreg/HR |  |  |  | HR | GCTA | Mreg | GCTA/HR | Mreg/HR |  |  |  | HR | GCTA | Mreg | GCTA/HR | Mreg/HR |
| Rsq=0.1 | 10K | 0.03 | 0.05 | 4.85 | 1.67 | 161.67 |  | Rsq=0.1 | 10K | 0.1 | 0.16 | 6.91 | 1.60 | 69.10 |  | Rsq=0.1 | 10K | 0.08 | 0.14 | 4.7 | 1.75 | 58.75 |
|  | 5K | 0.07 | 0.11 | 10.66 | 1.57 | 152.29 |  |  | 5K | 0.21 | 0.35 | 14.8 | 1.67 | 70.48 |  |  | 5K | 0.2 | 0.33 | 8.94 | 1.65 | 44.70 |
|  | 1k | 0.37 | 0.57 | 52.99 | 1.54 | 143.22 |  |  | 1k | 1.05 | 1.77 | 70.17 | 1.69 | 66.83 |  |  | 1k | 0.97 | 1.53 | 45.33 | 1.58 | 46.73 |
|  | 500 | 0.78 | 1.23 | 99.98 | 1.58 | 128.18 |  |  | 500 | 2.18 | 3.75 | 156.55 | 1.72 | 71.81 |  |  | 500 | 1.88 | 3.3 | 103.97 | 1.76 | 55.30 |
|  | 200 | 2.16 | 3.47 | 263.27 | 1.61 | 121.88 |  |  | 200 | 5.88 | 10.21 | 359.13 | 1.74 | 61.08 |  |  | 200 | 5.13 | 8.42 | 233.49 | 1.64 | 45.51 |
|  | 175 | 2.43 | 3.87 | 316.65 | 1.59 | 130.31 |  |  | 175 | 6.5 | 11.13 | 440.95 | 1.71 | 67.84 |  |  | 175 | 5.96 | 9.97 | 256.25 | 1.67 | 42.99 |
|  | 150 | 2.9 | 4.68 | 342.28 | 1.61 | 118.03 |  |  | 150 | 7.72 | 13.3 | 491.83 | 1.72 | 63.71 |  |  | 150 | 6.58 | 10.76 | 350.89 | 1.64 | 53.33 |
| Rsq=0.3 | 10K | 0.03 | 0.04 | 4.67 | 1.33 | 155.67 |  | Rsq=0.3 | 10K | 0.11 | 0.25 | 10.91 | 2.27 | 99.18 |  | Rsq=0.3 | 10K | 0.1 | 0.23 | 8.29 | 2.30 | 82.90 |
|  | 5K | 0.06 | 0.08 | 9.37 | 1.33 | 156.17 |  |  | 5K | 0.24 | 0.5 | 21.1 | 2.08 | 87.92 |  |  | 5K | 0.18 | 0.45 | 17.03 | 2.50 | 94.61 |
|  | 1k | 0.3 | 0.41 | 47.74 | 1.37 | 159.13 |  |  | 1k | 1.18 | 2.63 | 102.49 | 2.23 | 86.86 |  |  | 1k | 1.01 | 2.58 | 85.3 | 2.55 | 84.46 |
|  | 500 | 0.69 | 0.87 | 97.27 | 1.26 | 140.97 |  |  | 500 | 2.78 | 5.67 | 228.49 | 2.04 | 82.19 |  |  | 500 | 2.15 | 5.38 | 190.58 | 2.50 | 88.64 |
|  | 200 | 1.71 | 2.51 | 248.46 | 1.47 | 145.30 |  |  | 200 | 6.78 | 15.59 | 585.96 | 2.30 | 86.42 |  |  | 200 | 5.09 | 12.73 | 472.39 | 2.50 | 92.81 |
|  | 175 | 2.06 | 3.09 | 303.02 | 1.50 | 147.10 |  |  | 175 | 8.52 | 19.16 | 636.58 | 2.25 | 74.72 |  |  | 175 | 6.21 | 16.53 | 495.97 | 2.66 | 79.87 |
|  | 150 | 2.4 | 3.5 | 360.78 | 1.46 | 150.33 |  |  | 150 | 9.62 | 20.46 | 753.88 | 2.13 | 78.37 |  |  | 150 | 7.67 | 18.56 | 567.2 | 2.42 | 73.95 |
| Rsq=0.5 | 10K | 0.03 | 0.05 | 5.26 | 1.67 | 175.33 |  | Rsq=0.5 | 10K | 0.26 | 0.28 | 7.17 | 1.08 | 27.58 |  | Rsq=0.5 | 10K | 0.15 | 0.18 | 8.27 | 1.20 | 55.13 |
|  | 5K | 0.07 | 0.09 | 12.2 | 1.29 | 174.29 |  |  | 5K | 0.6 | 0.6 | 15.36 | 1.00 | 25.60 |  |  | 5K | 0.37 | 0.43 | 17.5 | 1.16 | 47.30 |
|  | 1k | 0.37 | 0.48 | 59.56 | 1.30 | 160.97 |  |  | 1k | 2.62 | 2.86 | 75.22 | 1.09 | 28.71 |  |  | 1k | 1.65 | 2.17 | 83.92 | 1.32 | 50.86 |
|  | 500 | 0.76 | 0.99 | 120.59 | 1.30 | 158.67 |  |  | 500 | 5.77 | 5.89 | 160.98 | 1.02 | 27.90 |  |  | 500 | 3.67 | 4.11 | 172.98 | 1.12 | 47.13 |
|  | 200 | 2.18 | 3.1 | 321.75 | 1.42 | 147.59 |  |  | 200 | 15.42 | 16.56 | 403.9 | 1.07 | 26.19 |  |  | 200 | 9.02 | 11.27 | 451.35 | 1.25 | 50.04 |
|  | 175 | 2.71 | 3.45 | 359.55 | 1.27 | 132.68 |  |  | 175 | 17.84 | 19.66 | 509.94 | 1.10 | 28.58 |  |  | 175 | 10.82 | 14.03 | 527.36 | 1.30 | 48.74 |
|  | 150 | 3.83 | 4.9 | 385.83 | 1.28 | 100.74 |  |  | 150 | 21.98 | 24.11 | 566.36 | 1.10 | 25.77 |  |  | 150 | 14.78 | 16.99 | 684.58 | 1.15 | 46.32 |
| Total | | 25.92 | 37.53 | 3426.73 | 30.42 | 3060.50 |  | Total | | 117.36 | 174.91 | 5618.68 | 34.61 | 1256.82 |  | Total | | 83.68 | 140.12 | 4786.29 | 37.61 | 1290.08 |
| Average | | 1.23 | 1.79 | 163.18 | 1.45 | 145.74 |  | Average | | 5.59 | 8.33 | 267.56 | 1.65 | 59.85 |  | Average | | 3.98 | 6.67 | 227.92 | 1.79 | 61.43 |

**A,** Mean square errors of HAPRAP, GCTA and multiple regression in 2-SNPs models; **B,** errors of HAPRAP, GCTA and multiple regression in 3-SNPs models. Rsq is the pair-wise linkage disequilibrium r^2^. N is the number of individuals in the genotypes reference panel. HR, Mreg and GCTA are the upper 95% CI of the errors of three methods separately. Increasing accuracy is the percentage of difference of error between HAPRAP and GCTA (or HAPRAP and Mulitple regression)

**Table S3.** Parametric simulation results

| N | log10(N) | MSE HAPRAP | RMSE_SD_HAPRAP | MSE COJO | RMSE_SD_COJO |
| --- | --- | --- | --- | --- | --- |
| 253288 | 5.403614615 | 0.028763948 | 0.008029695 | 0.020398702 | 0.011988 |
| 100000 | 5 | 0.038903313 | 0.009724 | 0.038231217 | 0.01021859 |
| 50000 | 4.698970004 | 0.041022793 | 0.01013654 | 0.045700224 | 0.0108118 |
| 10000 | 4 | 0.045569506 | 0.010164928 | 0.067732637 | 0.0109126 |
| 5000 | 3.698970004 | 0.064402474 | 0.0105237 | 0.114081209 | 0.011936 |
| 2500 | 3.397940009 | 0.079103536 | 0.0109473 | 0.176038685 | 0.012386 |
| 1750 | 3.243038049 | 0.101388704 | 0.011643 | 0.206827249 | 0.012859 |
| 1000 | 3 | 0.226729877 | 0.013187092 | 0.332479096 | 0.016987 |

MES is the mean square error between HAPRAP (GCTA-COJO) results and multiple regression results. SD is standard deviation. N is the sample size of meta-analysis. Log10(N) is the sample size in log scale.

**Table S4.** Performance Comparison of HAPRAP and GCTA Using BWHHS Summary Statistics and BWHHS Individual-level Genotypes.

| **BWHHS individual-level data** | | | | | | | | | | | | | | | | | | |
| --- | --- | --- | --- | --- | --- | --- | --- | --- | --- | --- | --- | --- | --- | --- | --- | --- | --- | --- |
|  | single SNP regression results | | | | |  | multiple regression results | | |  | HAPRAP results | | | |  | GCTA results | | |
| genomic regreions and traits | SNP | obs_BETA | obs_SE | obs_pavl | N |  | beta | SE | pval |  | beta (SHAPEIT) | beta (PLINK) | SE | pval |  | beta | SE | pval |
| NOS1AP_QTc | chr1:160291611 | 6.169 | 1.193 | 2.55E-07 | 1981 |  | 4.530 | 1.663 | 6.50E-03 |  | 4.221005781 | 4.368 | 1.747 | 1.25E-02 |  | 4.420 | 1.676 | 8.36E-03 |
| NOS1AP_QTc | chr1:160297312 | 3.235 | 0.840 | 1.21E-04 | 1980 |  | -2.122 | 1.822 | 2.44E-01 |  | -2.51980937 | -2.394 | 1.989 | 2.29E-01 |  | -1.976 | 1.807 | 2.74E-01 |
| NOS1AP_QTc | rs12143842 | 5.299 | 0.919 | 9.41E-09 | 1981 |  | 4.262 | 1.928 | 2.71E-02 |  | 4.590227142 | 4.583 | 2.119 | 3.06E-02 |  | 4.056 | 1.900 | 3.28E-02 |
| NOS1AP_QTc | rs16857031 | 3.098 | 1.111 | 5.36E-03 | 1981 |  | 2.815 | 1.591 | 7.70E-02 |  | 2.619420316 | 2.926 | 1.676 | 8.10E-02 |  | 2.797 | 1.595 | 7.95E-02 |
| NOS1AP_QTc | rs10918740 | 3.168 | 0.818 | 1.12E-04 | 1981 |  | -1.167 | 1.619 | 4.71E-01 |  | -0.9204972 | -1.202 | 1.721 | 4.85E-01 |  | -1.053 | 1.631 | 5.19E-01 |
| NOS1AP_QTc | chr1:160426106 | 5.868 | 1.242 | 2.49E-06 | 1981 |  | -0.817 | 2.945 | 7.82E-01 |  | -0.779834316 | -0.775 | 3.383 | 8.19E-01 |  | -0.408 | 2.817 | 8.85E-01 |
| NOS1AP_QTc | chr1:160427963 | 3.544 | 0.855 | 3.55E-05 | 1981 |  | 0.893 | 1.421 | 5.30E-01 |  | 0.672919201 | 0.715 | 1.597 | 6.54E-01 |  | 0.922 | 1.419 | 5.16E-01 |
| NOS1AP_QTc | chr1:160431793 | 4.938 | 1.103 | 8.04E-06 | 1981 |  | -0.610 | 2.413 | 8.01E-01 |  | -0.490834526 | -0.644 | 2.666 | 8.09E-01 |  | -0.793 | 2.362 | 7.37E-01 |
| NOS1AP_QTc | chr1:160435892 | 5.306 | 1.038 | 3.48E-07 | 1981 |  | 3.019 | 2.295 | 1.89E-01 |  | 2.969115271 | 3.135 | 2.662 | 2.39E-01 |  | 2.824 | 2.259 | 2.11E-01 |
| NOS1AP_QTc | chr1:160477234 | 4.295 | 0.898 | 1.86E-06 | 1981 |  | 2.069 | 1.773 | 2.43E-01 |  | 2.129289194 | 2.135 | 1.993 | 2.84E-01 |  | 2.047 | 1.758 | 2.44E-01 |
| NOS1AP_QTc | rs10919024 | 4.615 | 1.163 | 7.44E-05 | 1981 |  | -0.738 | 1.948 | 7.05E-01 |  | -0.788174598 | -0.861 | 2.137 | 6.87E-01 |  | -0.574 | 1.940 | 7.67E-01 |
| SCN5A10A_PR | chr3:38619266 | -3.379 | 0.977 | 5.53E-04 | 1896 |  | -2.881 | 0.983 | 3.41E-03 |  | -2.930359385 | -2.922 | 0.972 | 2.67E-03 |  | -2.953 | 0.987 | 2.77E-03 |
| SCN5A10A_PR | chr3:38634956 | 2.607 | 0.867 | 2.68E-03 | 1895 |  | 2.132 | 0.876 | 1.50E-02 |  | 2.073901206 | 2.123 | 0.862 | 1.39E-02 |  | 2.110 | 0.881 | 1.66E-02 |
| SCN5A10A_PR | chr3:38719374 | 2.460 | 0.814 | 2.55E-03 | 1895 |  | 0.707 | 1.040 | 4.96E-01 |  | 0.687633396 | 0.738 | 1.045 | 4.80E-01 |  | 0.735 | 1.046 | 4.83E-01 |
| SCN5A10A_PR | chr3:38742319 | 3.283 | 0.825 | 7.13E-05 | 1896 |  | 2.670 | 1.196 | 2.57E-02 |  | 2.754867715 | 2.719 | 1.210 | 2.48E-02 |  | 2.627 | 1.199 | 2.84E-02 |
| SCN5A10A_PR | chr3:38762595 | -2.398 | 0.824 | 3.64E-03 | 1894 |  | 0.075 | 1.156 | 9.48E-01 |  | 0.138299837 | 0.158 | 1.173 | 8.93E-01 |  | 0.040 | 1.162 | 9.72E-01 |
| LIPC_HDL | rs1077834 | 0.064 | 0.017 | 1.86E-04 | 1963 |  | 0.010 | 0.039 | 7.90E-01 |  | 0.02414668 | 0.023 | 0.039 | 5.49E-01 |  | 0.018 | 0.036 | 6.08E-01 |
| LIPC_HDL | rs8033940 | 0.055 | 0.015 | 3.45E-04 | 1963 |  | 0.017 | 0.028 | 5.44E-01 |  | 0.00908171 | 0.010 | 0.029 | 7.38E-01 |  | 0.014 | 0.027 | 6.04E-01 |
| LIPC_HDL | rs12914035 | -0.066 | 0.020 | 1.29E-03 | 1955 |  | -0.048 | 0.021 | 2.45E-02 |  | -0.0501866 | -0.050 | 0.021 | 1.73E-02 |  | -0.048 | 0.021 | 2.51E-02 |
| LIPC_HDL | rs261338 | 0.070 | 0.019 | 1.73E-04 | 1964 |  | 0.073 | 0.044 | 9.66E-02 |  | 0.06724443 | 0.065 | 0.046 | 1.54E-01 |  | 0.063 | 0.043 | 1.40E-01 |
| LIPC_HDL | rs261336 | 0.053 | 0.019 | 4.96E-03 | 1963 |  | -0.040 | 0.038 | 2.89E-01 |  | -0.040905 | -0.038 | 0.040 | 3.33E-01 |  | -0.035 | 0.038 | 3.52E-01 |
| LPL_HDL | chr8:19852310 | 0.041 | 0.021 | 4.45E-02 | 1963 |  | 0.002 | 0.035 | 9.57E-01 |  | 0.002991161 | 0.000 | 0.039 | 9.94E-01 |  | 0.006 | 0.034 | 8.63E-01 |
| LPL_HDL | chr8:19855067 | 0.046 | 0.027 | 8.27E-02 | 1964 |  | 0.046 | 0.044 | 2.98E-01 |  | 0.038682851 | 0.044 | 0.048 | 3.59E-01 |  | 0.034 | 0.043 | 4.28E-01 |
| LPL_HDL | chr8:19857947 | 0.060 | 0.019 | 1.58E-03 | 1963 |  | -0.009 | 0.038 | 8.07E-01 |  | -0.005405601 | -0.009 | 0.041 | 8.34E-01 |  | -0.004 | 0.037 | 9.14E-01 |
| LPL_HDL | chr8:19861214 | 0.035 | 0.017 | 4.01E-02 | 1963 |  | 0.018 | 0.047 | 6.92E-01 |  | 0.01801126 | 0.016 | 0.051 | 7.58E-01 |  | 0.006 | 0.046 | 8.95E-01 |
| LPL_HDL | chr8:19862322 | 0.027 | 0.016 | 8.48E-02 | 1964 |  | -0.061 | 0.036 | 8.84E-02 |  | -0.062664679 | -0.063 | 0.040 | 1.19E-01 |  | -0.053 | 0.035 | 1.31E-01 |
| LPL_HDL | chr8:19868772 | 0.050 | 0.016 | 1.60E-03 | 1963 |  | -0.011 | 0.046 | 8.11E-01 |  | -0.00779092 | -0.008 | 0.052 | 8.81E-01 |  | -0.003 | 0.045 | 9.43E-01 |
| LPL_HDL | rs1569209 | 0.029 | 0.027 | 2.95E-01 | 1962 |  | -0.159 | 0.067 | 1.76E-02 |  | -0.151147135 | -0.149 | 0.081 | 6.62E-02 |  | -0.134 | 0.062 | 3.07E-02 |
| LPL_HDL | rs10096633 | 0.076 | 0.022 | 7.17E-04 | 1964 |  | 0.206 | 0.059 | 5.30E-04 |  | 0.202920182 | 0.204 | 0.069 | 3.27E-03 |  | 0.188 | 0.057 | 9.22E-04 |
| LPL_HDL | chr8:19914551 | 0.050 | 0.016 | 2.54E-03 | 1964 |  | 0.041 | 0.047 | 3.86E-01 |  | 0.040571905 | 0.041 | 0.052 | 4.32E-01 |  | 0.039 | 0.047 | 4.13E-01 |
| LPL_HDL | chr8:19919284 | 0.051 | 0.014 | 3.78E-04 | 1963 |  | 0.041 | 0.020 | 4.30E-02 |  | 0.039647529 | 0.041 | 0.020 | 4.13E-02 |  | 0.040 | 0.020 | 4.81E-02 |
| LPL_HDL | chr8:19959564 | 0.009 | 0.016 | 5.69E-01 | 1952 |  | 0.007 | 0.022 | 7.62E-01 |  | 0.001014813 | -0.003 | 0.022 | 8.77E-01 |  | 0.004 | 0.022 | 8.52E-01 |
| LPL_HDL | rs9644568 | 0.036 | 0.022 | 9.96E-02 | 1964 |  | -0.013 | 0.037 | 7.34E-01 |  | -0.008263361 | -0.003 | 0.040 | 9.49E-01 |  | -0.006 | 0.036 | 8.65E-01 |
| LPL_HDL | chr8:19983329 | 0.009 | 0.028 | 7.53E-01 | 1964 |  | -0.063 | 0.046 | 1.66E-01 |  | -0.060358125 | -0.066 | 0.054 | 2.20E-01 |  | -0.070 | 0.045 | 1.22E-01 |
| LDLR_TC | rs6511720 | -0.059 | 0.060 | 3.31E-01 | 1967 |  | 0.171 | 0.105 | 1.02E-01 |  | 0.178254062 | 0.179 | 0.109 | 1.01E-01 |  | 0.170 | 0.104 | 1.03E-01 |
| LDLR_TC | chr19:11067575 | 0.019 | 0.038 | 6.25E-01 | 1967 |  | -0.036 | 0.047 | 4.47E-01 |  | -0.028491053 | -0.028 | 0.047 | 5.46E-01 |  | -0.026 | 0.046 | 5.70E-01 |
| LDLR_TC | chr19:11068516 | -0.140 | 0.097 | 1.47E-01 | 1968 |  | 0.021 | 0.125 | 8.69E-01 |  | 0.027147545 | 0.025 | 0.121 | 8.34E-01 |  | 0.020 | 0.122 | 8.67E-01 |
| LDLR_TC | rs2228671 | -0.139 | 0.059 | 1.79E-02 | 1967 |  | -0.314 | 0.129 | 1.52E-02 |  | -0.31271211 | -0.313 | 0.131 | 1.72E-02 |  | -0.300 | 0.126 | 1.74E-02 |
| LDLR_TC | rs2738447 | -0.006 | 0.039 | 8.76E-01 | 1937 |  | -0.033 | 0.052 | 5.20E-01 |  | -0.029060719 | -0.030 | 0.051 | 5.57E-01 |  | -0.027 | 0.051 | 6.00E-01 |
| LDLR_TC | rs5742911 | -0.044 | 0.042 | 2.91E-01 | 1967 |  | 0.016 | 0.056 | 7.73E-01 |  | 0.009234527 | 0.010 | 0.056 | 8.61E-01 |  | 0.008 | 0.055 | 8.82E-01 |
| SMARCA4_TC | rs8099996 | -0.039 | 0.040 | 3.27E-01 | 1968 |  | -0.044 | 0.085 | 6.04E-01 |  | -0.035465223 | -0.037 | 0.086 | 6.69E-01 |  | -0.034 | 0.083 | 6.84E-01 |
| SMARCA4_TC | rs8102273 | -0.034 | 0.041 | 4.05E-01 | 1967 |  | -0.050 | 0.118 | 6.70E-01 |  | -0.065980101 | -0.063 | 0.121 | 6.01E-01 |  | -0.067 | 0.116 | 5.62E-01 |
| SMARCA4_TC | chr19:11044837 | -0.016 | 0.044 | 7.21E-01 | 1968 |  | 0.095 | 0.095 | 3.20E-01 |  | 0.103250298 | 0.101 | 0.095 | 2.88E-01 |  | 0.104 | 0.095 | 2.73E-01 |
| SMARCA4_TC | chr19:11056030 | -0.036 | 0.049 | 4.58E-01 | 1968 |  | 0.041 | 0.079 | 6.02E-01 |  | 0.049370504 | 0.049 | 0.081 | 5.41E-01 |  | 0.047 | 0.078 | 5.49E-01 |
| SMARCA4_TC | chr19:11059187 | -0.071 | 0.061 | 2.46E-01 | 1967 |  | -0.116 | 0.104 | 2.66E-01 |  | -0.124172069 | -0.123 | 0.107 | 2.51E-01 |  | -0.121 | 0.103 | 2.41E-01 |
| PVRL2_TC | rs7254892 | -0.333 | 0.115 | 3.80E-03 | 1968 |  | -0.311 | 0.115 | 7.06E-03 |  | -0.312642 | -0.313 | 0.118 | 7.89E-03 |  | -0.317 | 0.115 | 6.01E-03 |
| PVRL2_TC | rs6857 | 0.124 | 0.052 | 1.62E-02 | 1961 |  | 0.115 | 0.052 | 2.58E-02 |  | 0.1129952 | 0.113 | 0.053 | 3.35E-02 |  | 0.115 | 0.052 | 2.59E-02 |
| APOB_TC | chr2:21079258 | -0.124 | 0.050 | 1.31E-02 | 1968 |  | -0.054 | 0.082 | 5.09E-01 |  | -0.035314534 | -0.045 | 0.080 | 5.71E-01 |  | -0.039 | 0.081 | 6.33E-01 |
| APOB_TC | chr2:21085700 | -0.104 | 0.039 | 8.33E-03 | 1968 |  | 0.048 | 0.085 | 5.77E-01 |  | 0.046720824 | 0.039 | 0.091 | 6.67E-01 |  | 0.043 | 0.081 | 5.95E-01 |
| APOB_TC | chr2:21091291 | -0.118 | 0.077 | 1.24E-01 | 1968 |  | 0.144 | 0.157 | 3.58E-01 |  | 0.100523751 | 0.082 | 0.189 | 6.63E-01 |  | 0.085 | 0.150 | 5.71E-01 |
| APOB_TC | rs41288783 | 0.743 | 0.464 | 1.09E-01 | 1968 |  | 0.760 | 0.463 | 1.01E-01 |  | 0.703382615 | 0.704 | 0.686 | 3.05E-01 |  | 0.766 | 0.467 | 1.01E-01 |
| APOB_TC | rs10199768 | 0.126 | 0.040 | 1.84E-03 | 1968 |  | -0.045 | 0.096 | 6.38E-01 |  | -0.033007134 | -0.034 | 0.105 | 7.43E-01 |  | -0.035 | 0.089 | 6.95E-01 |
| APOB_TC | chr2:21099811 | -0.175 | 0.074 | 1.81E-02 | 1968 |  | -0.185 | 0.186 | 3.21E-01 |  | -0.194506204 | -0.145 | 0.273 | 5.94E-01 |  | -0.128 | 0.169 | 4.49E-01 |
| APOB_TC | rs1367117 | 0.187 | 0.042 | 7.31E-06 | 1968 |  | 0.084 | 0.104 | 4.20E-01 |  | 0.085837786 | 0.096 | 0.130 | 4.59E-01 |  | 0.083 | 0.103 | 4.22E-01 |
| APOB_TC | chr2:21119728 | -0.160 | 0.172 | 3.52E-01 | 1968 |  | -0.040 | 0.214 | 8.51E-01 |  | 0.02447023 | 0.019 | 0.268 | 9.43E-01 |  | -0.035 | 0.209 | 8.66E-01 |
| APOB_TC | chr2:21120715 | -0.240 | 0.054 | 9.67E-06 | 1962 |  | -0.144 | 0.147 | 3.27E-01 |  | -0.110834464 | -0.121 | 0.224 | 5.91E-01 |  | -0.155 | 0.133 | 2.43E-01 |
| APOB_TC | rs17398765 | 0.249 | 0.070 | 4.15E-04 | 1968 |  | 0.186 | 0.088 | 3.52E-02 |  | 0.184781984 | 0.177 | 0.090 | 4.87E-02 |  | 0.186 | 0.088 | 3.54E-02 |
| APOB_TC | rs541041 | -0.198 | 0.051 | 9.80E-05 | 1967 |  | 0.004 | 0.142 | 9.76E-01 |  | -0.013999284 | -0.003 | 0.183 | 9.85E-01 |  | -0.008 | 0.135 | 9.55E-01 |
| APOB_TC | chr2:21158700 | 0.169 | 0.042 | 5.45E-05 | 1967 |  | 0.092 | 0.107 | 3.93E-01 |  | 0.0799806 | 0.047 | 0.123 | 7.04E-01 |  | 0.083 | 0.106 | 4.29E-01 |
| APOB_TC | chr2:21162840 | -0.257 | 0.071 | 3.15E-04 | 1967 |  | -0.059 | 0.179 | 7.42E-01 |  | -0.073469337 | -0.033 | 0.242 | 8.90E-01 |  | -0.039 | 0.167 | 8.16E-01 |
| APOB_TC | chr2:21181140 | -0.153 | 0.042 | 3.28E-04 | 1968 |  | 0.082 | 0.126 | 5.16E-01 |  | 0.054185813 | 0.015 | 0.171 | 9.30E-01 |  | 0.058 | 0.121 | 6.30E-01 |
| APOB_TC | chr2:21254166 | 0.119 | 0.041 | 3.29E-03 | 1967 |  | -0.078 | 0.103 | 4.51E-01 |  | -0.082762941 | -0.057 | 0.145 | 6.93E-01 |  | -0.072 | 0.099 | 4.66E-01 |
| APOB_TC | chr2:21257334 | -0.104 | 0.045 | 2.05E-02 | 1968 |  | -0.026 | 0.113 | 8.18E-01 |  | -0.013171934 | 0.001 | 0.146 | 9.96E-01 |  | -0.001 | 0.108 | 9.95E-01 |
| APOB_TC | chr2:21284866 | 0.160 | 0.045 | 3.79E-04 | 1960 |  | -0.076 | 0.121 | 5.30E-01 |  | -0.069744534 | -0.067 | 0.152 | 6.61E-01 |  | -0.089 | 0.115 | 4.42E-01 |
| APOB_TC | chr2:21300158 | -0.191 | 0.052 | 2.66E-04 | 1968 |  | -0.117 | 0.122 | 3.38E-01 |  | -0.106200392 | -0.084 | 0.156 | 5.92E-01 |  | -0.112 | 0.120 | 3.51E-01 |
| APOB_TC | chr2:21305109 | -0.077 | 0.050 | 1.27E-01 | 1968 |  | 0.164 | 0.109 | 1.33E-01 |  | 0.171493981 | 0.147 | 0.135 | 2.77E-01 |  | 0.159 | 0.104 | 1.25E-01 |
| APOB_TC | chr2:21305192 | -0.185 | 0.050 | 2.25E-04 | 1966 |  | -0.057 | 0.087 | 5.12E-01 |  | -0.050871902 | -0.077 | 0.089 | 3.89E-01 |  | -0.049 | 0.084 | 5.58E-01 |
| APOB_TC | chr2:21305332 | 0.168 | 0.041 | 4.45E-05 | 1966 |  | 0.090 | 0.109 | 4.10E-01 |  | 0.066201638 | 0.068 | 0.128 | 5.97E-01 |  | 0.088 | 0.101 | 3.85E-01 |
| APOB_TC | rs10198972 | -0.099 | 0.096 | 3.03E-01 | 1968 |  | 0.063 | 0.138 | 6.48E-01 |  | 0.061615785 | 0.089 | 0.208 | 6.68E-01 |  | 0.071 | 0.135 | 5.99E-01 |
| APOB_TC | rs312049 | 0.143 | 0.039 | 2.89E-04 | 1967 |  | 0.012 | 0.086 | 8.85E-01 |  | 0.032869696 | 0.031 | 0.102 | 7.58E-01 |  | 0.023 | 0.082 | 7.79E-01 |
| APOB_TC | rs7571647 | -0.243 | 0.063 | 1.14E-04 | 1968 |  | -0.084 | 0.112 | 4.54E-01 |  | -0.097097836 | -0.093 | 0.117 | 4.28E-01 |  | -0.097 | 0.109 | 3.74E-01 |
| PVRL2_LDL | rs6859 | 0.061 | 0.036 | 8.97E-02 | 1914 |  | 0.012 | 0.039 | 7.65E-01 |  | 0.0103051 | 0.012 | 0.038 | 7.51E-01 |  | 0.010 | 0.039 | 7.88E-01 |
| PVRL2_LDL | rs283813 | -0.113 | 0.069 | 9.96E-02 | 1911 |  | 0.066 | 0.090 | 4.61E-01 |  | 0.0713282 | 0.073 | 0.095 | 4.43E-01 |  | 0.067 | 0.090 | 4.56E-01 |
| PVRL2_LDL | rs7254892 | -0.464 | 0.104 | 9.18E-06 | 1915 |  | -0.503 | 0.135 | 1.92E-04 |  | -0.506265 | -0.509 | 0.144 | 4.16E-04 |  | -0.505 | 0.135 | 1.73E-04 |
| PVRL2_LDL | rs6857 | 0.151 | 0.047 | 1.36E-03 | 1909 |  | 0.126 | 0.051 | 1.44E-02 |  | 0.1216007 | 0.123 | 0.051 | 1.72E-02 |  | 0.126 | 0.052 | 1.45E-02 |
| TOMM40_LDL | rs157580 | -0.139 | 0.036 | 1.41E-04 | 1905 |  | -0.146 | 0.039 | 1.99E-04 |  | -0.146350702 | -0.146 | 0.038 | 1.13E-04 |  | -0.144 | 0.040 | 2.93E-04 |
| TOMM40_LDL | rs2075650 | 0.147 | 0.050 | 3.02E-03 | 1915 |  | 0.066 | 0.053 | 2.16E-01 |  | 0.06373579 | 0.064 | 0.054 | 2.36E-01 |  | 0.072 | 0.053 | 1.79E-01 |
| TOMM40_LDL | chr19:50095056 | 0.109 | 0.108 | 3.13E-01 | 1914 |  | 0.051 | 0.109 | 6.37E-01 |  | 0.044035751 | 0.045 | 0.111 | 6.87E-01 |  | 0.052 | 0.110 | 6.34E-01 |
| LDLR_LDL | rs6511720 | -0.061 | 0.055 | 2.63E-01 | 1914 |  | 0.163 | 0.094 | 8.25E-02 |  | 0.170090853 | 0.172 | 0.097 | 7.80E-02 |  | 0.164 | 0.094 | 8.08E-02 |
| LDLR_LDL | chr19:11068102 | -0.098 | 0.043 | 2.35E-02 | 1914 |  | -0.024 | 0.067 | 7.20E-01 |  | -0.036467497 | -0.036 | 0.066 | 5.89E-01 |  | -0.032 | 0.066 | 6.22E-01 |
| LDLR_LDL | rs2228671 | -0.142 | 0.053 | 7.89E-03 | 1914 |  | -0.267 | 0.112 | 1.74E-02 |  | -0.252933079 | -0.253 | 0.115 | 2.80E-02 |  | -0.248 | 0.111 | 2.54E-02 |
| LDLR_LDL | rs2738447 | -0.002 | 0.036 | 9.52E-01 | 1885 |  | -0.019 | 0.041 | 6.48E-01 |  | -0.018546278 | -0.018 | 0.042 | 6.75E-01 |  | -0.016 | 0.041 | 6.91E-01 |
| LDLR_LDL | rs5742911 | -0.037 | 0.038 | 3.23E-01 | 1914 |  | 0.019 | 0.048 | 6.95E-01 |  | 0.011975367 | 0.011 | 0.049 | 8.29E-01 |  | 0.011 | 0.048 | 8.25E-01 |
| SMARCA4_LDL | rs1529729 | 0.028 | 0.035 | 4.21E-01 | 1914 |  | 0.006 | 0.045 | 8.97E-01 |  | 0.004757544 | 0.005 | 0.046 | 9.11E-01 |  | 0.006 | 0.045 | 8.89E-01 |
| SMARCA4_LDL | rs3786725 | -0.041 | 0.038 | 2.80E-01 | 1915 |  | -0.052 | 0.084 | 5.36E-01 |  | -0.052182957 | -0.051 | 0.084 | 5.40E-01 |  | -0.053 | 0.085 | 5.29E-01 |
| SMARCA4_LDL | chr19:11044837 | -0.030 | 0.040 | 4.52E-01 | 1915 |  | 0.047 | 0.089 | 5.98E-01 |  | 0.045831229 | 0.045 | 0.087 | 6.03E-01 |  | 0.049 | 0.088 | 5.77E-01 |
| SMARCA4_LDL | chr19:11056030 | -0.060 | 0.044 | 1.80E-01 | 1915 |  | -0.008 | 0.074 | 9.15E-01 |  | -0.0073979 | -0.007 | 0.074 | 9.20E-01 |  | -0.008 | 0.072 | 9.15E-01 |
| SMARCA4_LDL | chr19:11059187 | -0.079 | 0.055 | 1.56E-01 | 1914 |  | -0.067 | 0.096 | 4.85E-01 |  | -0.069206839 | -0.069 | 0.097 | 4.78E-01 |  | -0.069 | 0.094 | 4.65E-01 |
| CELSR2_LDL | chr1:109600244 | -0.051 | 0.036 | 1.59E-01 | 1914 |  | 0.067 | 0.052 | 1.97E-01 |  | 0.063427672 | 0.066 | 0.052 | 2.11E-01 |  | 0.063 | 0.051 | 2.17E-01 |
| CELSR2_LDL | rs4970834 | -0.135 | 0.045 | 2.96E-03 | 1915 |  | 0.012 | 0.084 | 8.85E-01 |  | 0.015437984 | 0.016 | 0.086 | 8.48E-01 |  | 0.011 | 0.084 | 8.99E-01 |
| CELSR2_LDL | chr1:109618715 | -0.150 | 0.043 | 5.04E-04 | 1915 |  | -0.091 | 0.087 | 2.92E-01 |  | -0.095531199 | -0.095 | 0.090 | 2.91E-01 |  | -0.092 | 0.086 | 2.87E-01 |
| CELSR2_LDL | chr1:109618768 | -0.120 | 0.038 | 1.65E-03 | 1914 |  | -0.096 | 0.069 | 1.65E-01 |  | -0.0915356 | -0.094 | 0.071 | 1.85E-01 |  | -0.093 | 0.069 | 1.75E-01 |
| CELSR2_LDL | chr1:109622407 | -0.165 | 0.062 | 8.07E-03 | 1914 |  | -0.065 | 0.083 | 4.34E-01 |  | -0.069184414 | -0.070 | 0.081 | 3.87E-01 |  | -0.069 | 0.083 | 4.08E-01 |
| APOB_LDL | chr2:21079258 | -0.148 | 0.046 | 1.22E-03 | 1915 |  | -0.084 | 0.073 | 2.50E-01 |  | -0.073757686 | -0.088 | 0.077 | 2.52E-01 |  | -0.076 | 0.073 | 2.99E-01 |
| APOB_LDL | chr2:21085700 | -0.117 | 0.036 | 1.10E-03 | 1915 |  | 0.034 | 0.074 | 6.42E-01 |  | 0.045689045 | 0.045 | 0.077 | 5.59E-01 |  | 0.027 | 0.072 | 7.10E-01 |
| APOB_LDL | chr2:21093423 | 0.139 | 0.037 | 1.43E-04 | 1915 |  | -0.022 | 0.074 | 7.62E-01 |  | -0.006076934 | 0.003 | 0.076 | 9.67E-01 |  | -0.019 | 0.072 | 7.88E-01 |
| APOB_LDL | rs12713956 | -0.114 | 0.065 | 7.89E-02 | 1915 |  | 0.088 | 0.129 | 4.97E-01 |  | 0.040923764 | 0.046 | 0.174 | 7.91E-01 |  | 0.090 | 0.122 | 4.61E-01 |
| APOB_LDL | rs41288783 | 0.797 | 0.416 | 5.54E-02 | 1915 |  | 0.807 | 0.416 | 5.23E-02 |  | 0.720499481 | 0.715 | 0.493 | 1.47E-01 |  | 0.806 | 0.419 | 5.42E-02 |
| APOB_LDL | chr2:21103221 | -0.204 | 0.089 | 2.27E-02 | 1915 |  | 0.108 | 0.119 | 3.66E-01 |  | 0.088745387 | 0.100 | 0.134 | 4.56E-01 |  | 0.100 | 0.120 | 4.06E-01 |
| APOB_LDL | rs1367117 | 0.194 | 0.038 | 3.34E-07 | 1915 |  | 0.215 | 0.086 | 1.29E-02 |  | 0.213304679 | 0.198 | 0.096 | 3.81E-02 |  | 0.199 | 0.085 | 1.91E-02 |
| APOB_LDL | rs17398765 | 0.231 | 0.064 | 3.26E-04 | 1915 |  | 0.159 | 0.081 | 5.03E-02 |  | 0.151846195 | 0.149 | 0.084 | 7.42E-02 |  | 0.156 | 0.080 | 5.10E-02 |
| APOB_LDL | chr2:21124828 | -0.224 | 0.049 | 5.29E-06 | 1914 |  | -0.138 | 0.125 | 2.70E-01 |  | -0.102586799 | -0.099 | 0.180 | 5.85E-01 |  | -0.138 | 0.118 | 2.42E-01 |
| APOB_LDL | chr2:21162840 | -0.243 | 0.065 | 1.77E-04 | 1914 |  | 0.009 | 0.143 | 9.49E-01 |  | -0.014558366 | 0.001 | 0.199 | 9.98E-01 |  | 0.009 | 0.137 | 9.46E-01 |
| APOB_LDL | chr2:21167284 | 0.141 | 0.040 | 3.93E-04 | 1915 |  | -0.057 | 0.086 | 5.07E-01 |  | -0.064610131 | -0.050 | 0.095 | 5.95E-01 |  | -0.050 | 0.084 | 5.49E-01 |
| APOB_LDL | chr2:21181140 | -0.142 | 0.039 | 2.25E-04 | 1915 |  | 0.102 | 0.090 | 2.56E-01 |  | 0.090338433 | 0.079 | 0.117 | 5.01E-01 |  | 0.083 | 0.087 | 3.41E-01 |
| APOB_LDL | rs4635554 | 0.098 | 0.037 | 8.83E-03 | 1904 |  | -0.091 | 0.081 | 2.60E-01 |  | -0.087095985 | -0.072 | 0.110 | 5.13E-01 |  | -0.077 | 0.078 | 3.26E-01 |
| APOB_LDL | chr2:21257334 | -0.119 | 0.041 | 3.50E-03 | 1915 |  | -0.042 | 0.108 | 6.99E-01 |  | -0.060646945 | -0.049 | 0.128 | 7.02E-01 |  | -0.035 | 0.103 | 7.37E-01 |
| APOB_LDL | chr2:21292834 | -0.200 | 0.048 | 2.79E-05 | 1915 |  | -0.189 | 0.109 | 8.24E-02 |  | -0.165512721 | -0.163 | 0.135 | 2.28E-01 |  | -0.174 | 0.107 | 1.05E-01 |
| APOB_LDL | chr2:21303769 | 0.136 | 0.041 | 9.21E-04 | 1910 |  | 0.021 | 0.088 | 8.07E-01 |  | 0.025943413 | 0.009 | 0.119 | 9.40E-01 |  | 0.010 | 0.085 | 9.03E-01 |
| APOB_LDL | chr2:21305109 | -0.130 | 0.046 | 4.37E-03 | 1915 |  | 0.063 | 0.094 | 4.99E-01 |  | 0.061281757 | 0.066 | 0.112 | 5.52E-01 |  | 0.058 | 0.090 | 5.22E-01 |
| APOB_LDL | chr2:21305192 | -0.181 | 0.045 | 7.10E-05 | 1913 |  | -0.042 | 0.073 | 5.63E-01 |  | -0.034838403 | -0.055 | 0.072 | 4.42E-01 |  | -0.042 | 0.072 | 5.54E-01 |
| APOB_LDL | rs7571647 | -0.228 | 0.057 | 6.79E-05 | 1915 |  | -0.041 | 0.095 | 6.66E-01 |  | -0.038582137 | -0.039 | 0.111 | 7.24E-01 |  | -0.039 | 0.094 | 6.76E-01 |

**Table S5.** Summary of multiple associated SNPs at *ACAN*, *ADAAMTS17* and *PTCH1* for human height.

| **SNP** | **Nearest Gene** | **Effect Allele** | **Other Allele** | **GIANT Frequency (GIANT)** | **GIANT single regression** | | **GIANT joint SNP analysis** | |  | **HAPRAP** | | **COJO** | |
| --- | --- | --- | --- | --- | --- | --- | --- | --- | --- | --- | --- | --- | --- |
|  |  |  |  |  | **BETA** | **P-value** | **BETA** | **P-value** | **Frequency (ALSPAC)** | **BETA** | **P-value** | **BETA** | **P-value** |
| rs1348002 | *DET1* | C | G | 0.26 | 0.017 | 7.8E-08 | 0.020 | 1.5E-10 | 0.34 | 0.018 | 2.8E-09 | 0.018 | 6.4E-09 |
| rs11633371 | *ACAN* | T | G | 0.49 | 0.028 | 9.7E-21 | 0.024 | 2.1E-15 | 0.47 | 0.028 | 4.8E-20 | 0.028 | 1.1E-19 |
| rs16942341 | *ACAN* | T | C | 0.03 | -0.140 | 2.8E-43 | -0.114 | 3.0E-29 | 0.03 | -0.122 | 3.4E-34 | -0.124 | 3.9E-35 |
| rs2280470 | *ACAN* | A | G | 0.34 | 0.044 | 2.8E-44 | 0.031 | 5.5E-21 | 0.32 | 0.032 | 1.9E-25 | 0.040 | 1.1E-37 |
| rs3817428 | *ACAN* | C | G | 0.78 | 0.039 | 2.3E-31 | 0.022 | 2.6E-09 | 0.73 | 0.019 | 1.2E-08 | Under powered | Under powered |
| rs2238300 | *FANCI* | A | G | 0.35 | -0.021 | 2.3E-12 | -0.018 | 1.6E-09 | 0.38 | -0.020 | 3.8E-11 | -0.020 | 1.8E-11 |
| rs2573625 | *ADAMTS17* | T | C | 0.65 | 0.029 | 5.7E-20 | 0.030 | 3.7E-22 | 0.33 | 0.025 | 2.4E-15 | 0.031 | 4.5E-23 |
| rs1529889 | *ADAMTS17* | A | G | 0.46 | 0.026 | 6.2E-17 | non-significant | non-significant | 0.46 | 0.019 | 6.4E-10 | Under powered | Under powered |
| rs4246302 | *ADAMTS17* | A | G | 0.67 | -0.027 | 1.7E-16 | -0.027 | 1.4E-16 | 0.31 | -0.028 | 1.4E-17 | -0.028 | 1.0E-17 |
| rs4548838 | *ADAMTS17* | T | C | 0.45 | 0.033 | 8.6E-28 | 0.034 | 9.1E-30 | 0.45 | 0.033 | 1.4E-28 | 0.033 | 1.7E-28 |
| rs7170986 | *LRRK1* | A | G | 0.21 | -0.019 | 6.0E-08 | -0.019 | 1.1E-08 | 0.22 | -0.018 | 4.5E-08 | Under powered | Under powered |
| rs8042424 | *CHSY1* | T | C | 0.27 | -0.023 | 7.9E-11 | -0.022 | 5.1E-10 | 0.24 | -0.022 | 2.2E-10 | -0.022 | 2.0E-10 |
| rs1257763 | *PTPDC1* | A | G | 0.03 | 0.078 | 1.9E-16 | 0.071 | 9.4E-14 | 0.04 | 0.067 | 2.2E-12 | 0.069 | 3.0E-13 |
| rs12347744 | *C9orf3* | T | C | 0.03 | -0.039 | 4.7E-11 | -0.056 | 2.8E-20 | 0.07 | -0.054 | 1.7E-19 | -0.048 | 2.0E-15 |
| rs357564 | *PTCH1* | A | G | 0.33 | -0.046 | 6.1E-22 | non-significant | non-significant | 0.33 | -0.034 | 3.9E-13 | Under powered | Under powered |
| rs4448343 | *PTCH1* | A | G | 0.68 | -0.035 | 4.5E-30 | -0.035 | 1.1E-28 | 0.34 | -0.026 | 2.0E-17 | -0.032 | 3.9E-24 |
| rs1329393 | *PTCH1* | T | C | 0.13 | 0.034 | 2.4E-13 | 0.038 | 1.4E-15 | 0.17 | 0.034 | 5.1E-13 | 0.030 | 2.5E-10 |
| rs817300 | *PTCH1* | A | G | 0.04 | -0.085 | 4.3E-34 | -0.070 | 2.2E-23 | 0.07 | -0.056 | 4.8E-16 | -0.061 | 1.6E-18 |
| rs10990303 | *PTCH1* | T | C | 0.23 | 0.036 | 4.4E-24 | 0.032 | 1.4E-19 | 0.22 | 0.030 | 5.4E-18 | 0.028 | 8.4E-16 |
| rs7870753 | *HABP4* | A | G | 0.78 | -0.043 | 3.5E-33 | -0.045 | 1.7E-37 | 0.22 | -0.040 | 1.3E-30 | -0.041 | 2.9E-31 |

**Table S6.** R^2^ between the novel identified independent SNPs and nearby SNPs

| CHR_A | BP_A | SNP_A |  | CHR_B | BP_B | SNP_B | R2 |
| --- | --- | --- | --- | --- | --- | --- | --- |
| 9 | 98209594 | rs357564 |  | 9 | 98266370 | rs4448343 | 0.212333 |
| 9 | 98209594 | rs357564 |  | 9 | 98318926 | rs1329393 | 0.00304903 |
| 9 | 98209594 | rs357564 |  | 9 | 98380222 | rs817300 | 0.0608457 |
| 9 | 98209594 | rs357564 |  | 9 | 98410405 | rs10990303 | 0.0017851 |
| 9 | 98209594 | rs357564 |  | 9 | 99201585 | rs7870753 | 0.00182932 |
| 15 | 100539258 | rs1529889 |  | 15 | 100687967 | rs4246302 | 0.000168859 |
| 15 | 100539258 | rs1529889 |  | 15 | 100761190 | rs4548838 | 2.51479e-05 |

**Table S7.** Performance Comparison of HAPRAP, GCTA and Multiple Regression Using a Simulated Population with Two Rare Variants.

| SNP1 | | | | | | |
| --- | --- | --- | --- | --- | --- | --- |
|  | Rsq=0.9 | | | | | |
|  | HR_MEAN | HR_SD | Mreg_MEAN | Mreg_SD | GCTA_MEAN | GCTA_SD |
| 10K | 0.002579 | 0.011049 | 0.000874 | 0.239421 | 0.003993 | 0.03148 |
| 5K | 0.003626 | 0.015617 | 0.023512 | 0.344624 | 0.001064 | 0.04715 |
| 1k | 0.005342 | 0.038019 | 0.002425 | 0.804111 | 0.010605 | 0.112993 |
| 500 | 0.014124 | 0.055718 | 0.001902 | 1.15045 | 0.052179 | 0.217501 |
| 100 | 0.014404 | 0.122847 | 0.069349 | 2.682938 | 0.057968 | 0.292097 |
| SNP2 | | | | | | |
|  | Rsq=0.9 | | | | | |
|  | HR_MEAN | HR_SD | Mreg_MEAN | Mreg_SD | GCTA_MEAN | GCTA_SD |
| 10K | 0.002847 | 0.012574 | 0.001868 | 0.249512 | 0.004604 | 0.03517 |
| 5K | 0.003952 | 0.017793 | 0.020453 | 0.359565 | 0.000881 | 0.052533 |
| 1k | 0.005489 | 0.043123 | 0.012581 | 0.843051 | 0.01035 | 0.124852 |
| 500 | 0.014384 | 0.062825 | 0.013464 | 1.204385 | 0.054212 | 0.237283 |
| 100 | 0.013037 | 0.154193 | 0.050376 | 2.783836 | 0.054051 | 0.324903 |

We assumed that SNP1 has an independent SNP effect of 1 and SNP2 is a bystander SNP without own effect. The minor allele frequencies of SNP1 and SNP2 are 0.093 and 0.083 respectively. The pair-wise linkage disequilibrium (r^2^) between the two SNPs is 0.9. Rsq is the pair-wise linkage disequilibrium r^2^. N is the number of individuals in the genotypes reference panel. HR_MEAN (and HR_SD) are the means (and standard deviations (SD)) for differences between HAPRAP betas and multiple regression joint SNP effects. Mreg_MEAN (and Mreg_SD) are the means (and SDs) for differences between multiple regression joint SNP effects of the reference panel and multiple regression joint SNP effects of the whole population. GCTA_MEAN (and GCTA_SD) are the means (and SDs) for differences between GCTA betas and multiple regression joint SNP effects.

**Table S8.** Performance Comparison of HAPRAP and GCTA Using BWHHS Summary Statistics and 1000 Genome Individual-Level Genotypes.

| **BWHHS summary data with the 1000 Genome individual-level data** | | | | | | | | | | | | | | | | | | |
| --- | --- | --- | --- | --- | --- | --- | --- | --- | --- | --- | --- | --- | --- | --- | --- | --- | --- | --- |
| single SNP analysis | | | | | |  | HAPRAP | | | |  | Mreg | | |  | GCTA | | |
| Traits | SNP | obs_beta | obs_SE | obs_pavl | N |  | beta  (SHAPEIT) | beta  (PLINK) | SE | pval |  | beta | SE | pval |  | beta | SE | pval |
| LPL_HDL | rs74304285 | 0.041 | 0.021 | 0.045 | 1963 |  | -0.028 | -0.028 | 0.043 | 0.513 |  | -0.001 | 0.034 | 0.976 |  | -0.024 | 0.042 | 0.570 |
| LPL_HDL | rs343 | 0.046 | 0.027 | 0.083 | 1964 |  | -0.001 | -0.001 | 0.046 | 0.977 |  | 0.001 | 0.040 | 0.988 |  | 0.059 | 0.049 | 0.226 |
| LPL_HDL | rs269 | 0.060 | 0.019 | 0.002 | 1963 |  | 0.024 | 0.024 | 0.045 | 0.594 |  | 0.022 | 0.035 | 0.544 |  | -0.006 | 0.042 | 0.889 |
| LPL_HDL | rs301 | 0.035 | 0.017 | 0.040 | 1963 |  | 0.008 | 0.008 | 0.020 | 0.708 |  | -0.013 | 0.045 | 0.775 |  | 0.001 | 0.023 | 0.961 |
| LPL_HDL | rs314 | 0.027 | 0.016 | 0.085 | 1964 |  | -0.079 | -0.079 | 0.032 | 0.013 |  | -0.040 | 0.035 | 0.251 |  | -0.087 | 0.032 | 0.007 |
| LPL_HDL | rs13702 | 0.050 | 0.016 | 0.002 | 1963 |  | -0.013 | -0.013 | 0.050 | 0.791 |  | 0.014 | 0.044 | 0.745 |  | -0.026 | 0.046 | 0.566 |
| LPL_HDL | rs10096633 | 0.076 | 0.022 | 0.001 | 1964 |  | 0.149 | 0.149 | 0.044 | 0.001 |  | 0.122 | 0.047 | 0.009 |  | 0.249 | 0.062 | 0.000 |
| LPL_HDL | rs35237252 | 0.050 | 0.016 | 0.003 | 1964 |  | 0.065 | 0.065 | 0.047 | 0.165 |  | 0.025 | 0.046 | 0.580 |  | 0.108 | 0.048 | 0.025 |
| LPL_HDL | rs4333617 | 0.051 | 0.014 | 0.000 | 1963 |  | 0.045 | 0.045 | 0.020 | 0.022 |  | 0.038 | 0.019 | 0.047 |  | 0.029 | 0.019 | 0.125 |
| LPL_HDL | rs9644568 | 0.036 | 0.022 | 0.100 | 1964 |  | -0.010 | -0.010 | 0.036 | 0.776 |  | -0.012 | 0.034 | 0.728 |  | -0.040 | 0.044 | 0.364 |
| LPL_HDL | rs7015766 | 0.009 | 0.028 | 0.753 | 1964 |  | -0.113 | -0.113 | 0.043 | 0.009 |  | -0.105 | 0.042 | 0.013 |  | -0.232 | 0.061 | 0.000 |
| LIPC_HDL | rs12914035 | -0.066 | 0.020 | 0.001 | 1955 |  | -0.054 | -0.054 | 0.020 | 0.008 |  | -0.051 | 0.021 | 0.014 |  | -0.054 | 0.021 | 0.009 |
| LIPC_HDL | rs261338 | 0.070 | 0.019 | 0.000 | 1964 |  | 0.060 | 0.060 | 0.020 | 0.002 |  | 0.059 | 0.019 | 0.002 |  | 0.062 | 0.019 | 0.001 |
| HERPUD1_HDL | rs2562126 | -0.021 | 0.021 | 0.309 | 1963 |  | 0.007 | 0.011 | 0.022 | 0.606 |  | 0.005 | 0.022 | 0.810 |  | 0.012 | 0.022 | 0.595 |
| HERPUD1_HDL | rs9938160 | 0.022 | 0.015 | 0.160 | 1964 |  | -0.015 | -0.019 | 0.017 | 0.279 |  | -0.012 | 0.016 | 0.467 |  | -0.019 | 0.017 | 0.265 |
| HERPUD1_HDL | rs7205692 | -0.046 | 0.020 | 0.020 | 1964 |  | -0.042 | -0.044 | 0.023 | 0.052 |  | -0.035 | 0.023 | 0.127 |  | -0.029 | 0.024 | 0.229 |
| HERPUD1_HDL | rs12920974 | -0.077 | 0.016 | 1.38E-06 | 1963 |  | -0.057 | -0.058 | 0.020 | 0.005 |  | -0.056 | 0.020 | 0.005 |  | -0.051 | 0.019 | 0.008 |
| HERPUD1_HDL | rs3764261 | 0.103 | 0.015 | 2.29E-11 | 1963 |  | 0.074 | 0.075 | 0.021 | 0.000 |  | 0.077 | 0.020 | 0.000 |  | 0.084 | 0.020 | 0.000 |
| CETP_HDL | rs1864163 | -0.105 | 0.016 | 1.71E-10 | 1964 |  | -0.045 | -0.047 | 0.028 | 0.092 |  | -0.054 | 0.024 | 0.026 |  | -0.051 | 0.027 | 0.059 |
| CETP_HDL | rs118146573 | -0.098 | 0.022 | 5.60E-06 | 1964 |  | -0.062 | -0.075 | 0.036 | 0.035 |  | -0.056 | 0.032 | 0.085 |  | -0.067 | 0.035 | 0.052 |
| CETP_HDL | rs11076174 | -0.104 | 0.025 | 4.59E-05 | 1964 |  | -0.078 | -0.079 | 0.032 | 0.014 |  | -0.082 | 0.029 | 0.005 |  | -0.064 | 0.032 | 0.042 |
| CETP_HDL | rs5883 | 0.118 | 0.031 | 0.000 | 1962 |  | 0.091 | 0.089 | 0.029 | 0.002 |  | 0.090 | 0.032 | 0.004 |  | 0.070 | 0.033 | 0.033 |
| CETP_HDL | rs289715 | 0.038 | 0.022 | 0.079 | 1964 |  | 0.022 | 0.010 | 0.025 | 0.692 |  | 0.014 | 0.022 | 0.532 |  | 0.011 | 0.022 | 0.630 |
| CETP_HDL | rs5880 | -0.058 | 0.031 | 0.062 | 1964 |  | 0.006 | 0.036 | 0.034 | 0.280 |  | 0.001 | 0.034 | 0.983 |  | 0.040 | 0.038 | 0.295 |
| PVRL2_LDL | rs7254892 | -0.464 | 0.104 | 9.18E-06 | 1915 |  | -0.443 | -0.443 | 0.101 | 1.29E-05 |  | -0.444 | 0.105 | 0.000 |  | -0.424 | 0.106 | 0.000 |
| PVRL2_LDL | rs6857 | 0.151 | 0.047 | 0.001 | 1909 |  | 0.134 | 0.134 | 0.049 | 0.006 |  | 0.138 | 0.047 | 0.003 |  | 0.123 | 0.048 | 0.010 |
| LDLR_LDL | rs2228671 | -0.142 | 0.053 | 0.008 | 1914 |  | -0.137 | -0.137 | 0.066 | 0.037 |  | -0.150 | 0.059 | 0.011 |  | -0.140 | 0.057 | 0.014 |
| LDLR_LDL | rs5742911 | -0.037 | 0.038 | 0.323 | 1914 |  | -0.011 | -0.010 | 0.042 | 0.807 |  | 0.012 | 0.042 | 0.776 |  | -0.004 | 0.040 | 0.929 |
| SMARCA4_LDL | rs3786725 | -0.041 | 0.038 | 0.280 | 1915 |  | -0.021 | -0.021 | 0.042 | 0.615 |  | -0.019 | 0.043 | 0.647 |  | -0.023 | 0.041 | 0.575 |
| SMARCA4_LDL | rs11668477 | -0.060 | 0.044 | 0.180 | 1915 |  | -0.034 | -0.034 | 0.056 | 0.545 |  | -0.026 | 0.067 | 0.701 |  | -0.027 | 0.057 | 0.629 |
| SMARCA4_LDL | rs17248720 | -0.079 | 0.055 | 0.156 | 1914 |  | -0.037 | -0.037 | 0.082 | 0.651 |  | -0.043 | 0.084 | 0.613 |  | -0.048 | 0.070 | 0.492 |
| CELSR2_LDL | rs7528419 | -0.150 | 0.043 | 0.001 | 1915 |  | -0.127 | -0.127 | 0.054 | 0.018 |  | -0.125 | 0.053 | 0.018 |  | -0.124 | 0.050 | 0.013 |
| CELSR2_LDL | rs11577931 | -0.165 | 0.062 | 0.008 | 1914 |  | -0.056 | -0.056 | 0.080 | 0.484 |  | -0.060 | 0.076 | 0.429 |  | -0.074 | 0.072 | 0.303 |
| APOB_LDL | rs41288783 | 0.797 | 0.416 | 0.055 | 1915 |  | 0.705 | 0.705 | 0.206 | 0.001 |  | 0.731 | 0.413 | 0.077 |  | 0.449 | 0.429 | 0.296 |
| APOB_LDL | rs1367117 | 0.194 | 0.038 | 3.34E-07 | 1915 |  | 0.103 | 0.103 | 0.046 | 0.024 |  | 0.126 | 0.043 | 0.004 |  | 0.119 | 0.043 | 0.006 |
| APOB_LDL | rs17398765 | 0.231 | 0.064 | 0.000 | 1915 |  | 0.131 | 0.131 | 0.080 | 0.100 |  | 0.114 | 0.070 | 0.105 |  | 0.143 | 0.069 | 0.039 |
| APOB_LDL | rs1713222 | -0.224 | 0.049 | 5.29E-06 | 1914 |  | -0.164 | -0.164 | 0.049 | 0.001 |  | -0.159 | 0.051 | 0.002 |  | -0.161 | 0.052 | 0.002 |
| NOS1AP_QTc | rs10429888 | 6.169 | 1.193 | 2.55E-07 | 1981 |  | 4.134 | 4.297 | 1.739 | 0.014 |  | 4.084 | 1.639 | 0.013 |  | 4.289 | 1.740 | 0.014 |
| NOS1AP_QTc | rs12143842 | 5.299 | 0.919 | 9.41E-09 | 1981 |  | 1.591 | 1.610 | 1.441 | 0.264 |  | 1.586 | 1.332 | 0.234 |  | 1.904 | 1.368 | 0.164 |
| NOS1AP_QTc | rs16857031 | 3.098 | 1.111 | 0.005 | 1981 |  | 2.579 | 2.434 | 1.388 | 0.080 |  | 1.985 | 1.294 | 0.125 |  | 2.390 | 1.271 | 0.060 |
| NOS1AP_QTc | rs4657166 | 3.544 | 0.855 | 3.55E-05 | 1981 |  | 1.131 | 1.347 | 1.084 | 0.214 |  | 1.160 | 0.992 | 0.242 |  | 1.272 | 1.030 | 0.217 |
| NOS1AP_QTc | rs10918859 | 5.306 | 1.038 | 3.48E-07 | 1981 |  | 3.445 | 3.234 | 1.201 | 0.007 |  | 3.277 | 1.152 | 0.005 |  | 3.661 | 1.179 | 0.002 |
| SCN5A10A_PR | rs62242769 | -3.379 | 0.977 | 0.001 | 1896 |  | -3.204 | -3.450 | 1.064 | 0.001 |  | -2.931 | 0.979 | 0.003 |  | -3.197 | 0.983 | 0.001 |
| SCN5A10A_PR | rs55824920 | 2.607 | 0.867 | 0.003 | 1895 |  | 2.272 | 2.088 | 0.872 | 0.017 |  | 2.171 | 0.868 | 0.012 |  | 2.048 | 0.876 | 0.019 |
| SCN5A10A_PR | rs6801957 | 3.283 | 0.825 | 7.13E-05 | 1896 |  | 3.148 | 3.244 | 0.851 | 0.000 |  | 3.061 | 0.824 | 0.000 |  | 3.106 | 0.832 | 0.000 |
| LDLR_TC | rs6511720 | -0.059 | 0.060 | 0.331 | 1967 |  | 0.179 | 0.237 | 0.124 | 0.056 |  | 0.174 | 0.103 | 0.092 |  | 0.196 | 0.108 | 0.071 |
| LDLR_TC | rs6511721 | 0.019 | 0.038 | 0.625 | 1967 |  | -0.006 | -0.014 | 0.046 | 0.754 |  | -0.027 | 0.045 | 0.547 |  | -0.021 | 0.047 | 0.656 |
| LDLR_TC | rs73015030 | -0.140 | 0.097 | 0.147 | 1968 |  | -0.040 | 0.014 | 0.138 | 0.917 |  | 0.025 | 0.121 | 0.836 |  | 0.034 | 0.125 | 0.788 |
| LDLR_TC | rs2228671 | -0.139 | 0.059 | 0.018 | 1967 |  | -0.267 | -0.343 | 0.134 | 0.011 |  | -0.307 | 0.125 | 0.014 |  | -0.310 | 0.126 | 0.014 |
| LDLR_TC | rs2738447 | -0.006 | 0.039 | 0.876 | 1937 |  | 0.004 | -0.003 | 0.049 | 0.945 |  | -0.029 | 0.051 | 0.574 |  | -0.009 | 0.053 | 0.863 |
| LDLR_TC | rs5742911 | -0.044 | 0.042 | 0.291 | 1967 |  | -0.025 | -0.023 | 0.051 | 0.655 |  | 0.010 | 0.054 | 0.854 |  | -0.016 | 0.052 | 0.752 |
| SMARCA4_TC | rs8099996 | -0.039 | 0.040 | 0.327 | 1968 |  | -0.037 | -0.033 | 0.083 | 0.687 |  | -0.044 | 0.085 | 0.604 |  | -0.032 | 0.087 | 0.711 |
| SMARCA4_TC | rs8102273 | -0.034 | 0.041 | 0.405 | 1967 |  | -0.060 | -0.066 | 0.115 | 0.564 |  | -0.050 | 0.118 | 0.670 |  | -0.074 | 0.119 | 0.535 |
| SMARCA4_TC | rs73015007 | -0.016 | 0.044 | 0.721 | 1968 |  | 0.094 | 0.096 | 0.098 | 0.331 |  | 0.095 | 0.095 | 0.320 |  | 0.105 | 0.097 | 0.279 |
| SMARCA4_TC | rs11668477 | -0.036 | 0.049 | 0.458 | 1968 |  | 0.037 | 0.041 | 0.075 | 0.587 |  | 0.041 | 0.079 | 0.602 |  | 0.045 | 0.075 | 0.543 |
| SMARCA4_TC | rs17248720 | -0.071 | 0.061 | 0.246 | 1967 |  | -0.109 | -0.111 | 0.108 | 0.306 |  | -0.116 | 0.104 | 0.266 |  | -0.115 | 0.094 | 0.221 |
| PVRL2_TC | rs7254892 | -0.333 | 0.115 | 0.004 | 1968 |  | -0.315 | -0.315 | 0.113 | 0.005 |  | -0.311 | 0.115 | 0.007 |  | -0.299 | 0.116 | 0.010 |
| PVRL2_TC | rs6857 | 0.124 | 0.052 | 0.016 | 1961 |  | 0.112 | 0.112 | 0.054 | 0.037 |  | 0.115 | 0.052 | 0.026 |  | 0.104 | 0.052 | 0.046 |
| APOB_TC | rs1367117 | 0.187 | 0.042 | 7.31E-06 | 1968 |  | 0.108 | 0.108 | 0.049 | 0.027 |  | 0.109 | 0.048 | 0.022 |  | 0.113 | 0.046 | 0.015 |
| APOB_TC | rs1800481 | -0.240 | 0.054 | 9.67E-06 | 1962 |  | -0.186 | -0.186 | 0.053 | 0.000 |  | -0.181 | 0.057 | 0.001 |  | -0.182 | 0.057 | 0.001 |
| APOB_TC | rs17398765 | 0.249 | 0.070 | 0.000 | 1968 |  | 0.134 | 0.134 | 0.083 | 0.106 |  | 0.142 | 0.077 | 0.067 |  | 0.158 | 0.075 | 0.036 |

**Table S9.** The two-way ANOVA of testing the difference of mean of multiple regression, HAPRAP (PLINK), HAPRAP (SHAPEIT) and GCTA-COJO

|  | Df | SumSq | MeanSq | Fvalue | Pr(>F) |
| --- | --- | --- | --- | --- | --- |
| Method | 3 | 0.03 | 0.0102 | 0.0033 | 0.99973 |
| Trait | 4 | 41.47 | 10.3679 | 3.3976 | 0.009404 |
| Method:Trait | 12 | 0.05 | 0.0044 | 0.0015 | 1 |

Notation: Df refers to degree of freedom. SumSq is the sum of square, MeanSq is the mean of square. Fvalue is the statistic of the F test. Pr is the p value of the F test.

**Table S10.** The residuals of t-statistics of HAPRAP and GCTA-COJO

| Traits | SNP | T_Mreg | T_HS | T_HP | T_GCTA | R_HS | R_HP | R_G |
| --- | --- | --- | --- | --- | --- | --- | --- | --- |
| LPL_HDL | rs74304285 | -0.03060766 | -0.654702279 | -0.654681466 | -0.568250427 | 0.389494093 | 0.389468115 | 0.289059745 |
| LPL_HDL | rs343 | 0.014512929 | -0.028257226 | -0.028259652 | 1.211535531 | 0.001829286 | 0.001829494 | 1.432863108 |
| LPL_HDL | rs269 | 0.60604003 | 0.532774761 | 0.532731403 | -0.139007523 | 0.0053678 | 0.005374155 | 0.555095856 |
| LPL_HDL | rs301 | -0.286343661 | 0.374350703 | 0.374390692 | 0.048944354 | 0.436517043 | 0.436569886 | 0.112418053 |
| LPL_HDL | rs314 | -1.147270316 | -2.48709351 | -2.487184767 | -2.710318163 | 1.795126191 | 1.795370736 | 2.443118572 |
| LPL_HDL | rs13702 | 0.324786791 | -0.265281385 | -0.265275678 | -0.574322609 | 0.348180452 | 0.348173717 | 0.808397712 |
| LPL_HDL | rs10096633 | 2.600933187 | 3.372452161 | 3.372547678 | 3.996800108 | 0.595241528 | 0.595388923 | 1.94844446 |
| LPL_HDL | rs35237252 | 0.554036025 | 1.389408675 | 1.389435859 | 2.236839097 | 0.697847463 | 0.697892883 | 2.831826179 |
| LPL_HDL | rs4333617 | 1.990585958 | 2.300382449 | 2.300366844 | 1.532931198 | 0.095973866 | 0.095964197 | 0.209447879 |
| LPL_HDL | rs9644568 | -0.347845151 | -0.284696339 | -0.284770069 | -0.907280846 | 0.003987772 | 0.003978466 | 0.312968296 |
| LPL_HDL | rs7015766 | -2.494142138 | -2.625368016 | -2.625370052 | -3.83065389 | 0.017220231 | 0.017220765 | 1.786263663 |
| LIPC_HDL | rs12914035 | -2.46685879 | -2.660052533 | -2.6601947 | -2.628283053 | 0.037323823 | 0.037378774 | 0.026057793 |
| LIPC_HDL | rs261338 | 3.113029827 | 3.031157676 | 3.031151503 | 3.261197381 | 0.006703049 | 0.00670406 | 0.021953624 |
| HERPUD1_HDL | rs2562126 | 0.240772158 | 0.304071597 | 0.515907962 | 0.53205949 | 0.004006819 | 0.07569971 | 0.08484831 |
| HERPUD1_HDL | rs9938160 | -0.727759523 | -0.888647845 | -1.083363982 | -1.114194978 | 0.025885052 | 0.126454531 | 0.149332361 |
| HERPUD1_HDL | rs7205692 | -1.52640883 | -1.862479615 | -1.94338722 | -1.202700434 | 0.112943573 | 0.173870978 | 0.104787126 |
| HERPUD1_HDL | rs12920974 | -2.812118152 | -2.797890106 | -2.838245378 | -2.633310208 | 0.000202437 | 0.000682632 | 0.031972281 |
| HERPUD1_HDL | rs3764261 | 3.848325311 | 3.434745868 | 3.496121607 | 4.129374997 | 0.171047956 | 0.124047449 | 0.078988926 |
| CETP_HDL | rs1864163 | -2.234384638 | -1.603461077 | -1.686576708 | -1.891561576 | 0.39806454 | 0.300093528 | 0.117527651 |
| CETP_HDL | rs118146573 | -1.721622204 | -1.745426074 | -2.105503618 | -1.943844017 | 0.000566624 | 0.147364941 | 0.049382534 |
| CETP_HDL | rs11076174 | -2.826542975 | -2.431725766 | -2.44857562 | -2.032264808 | 0.155880628 | 0.142859321 | 0.630877806 |
| CETP_HDL | rs5883 | 2.861702802 | 3.120448649 | 3.041041512 | 2.130016758 | 0.066949413 | 0.032162373 | 0.535364467 |
| CETP_HDL | rs289715 | 0.624840938 | 0.879736721 | 0.395652881 | 0.48191705 | 0.06497186 | 0.052527165 | 0.020427238 |
| CETP_HDL | rs5880 | 0.021184345 | 0.178252083 | 1.080394372 | 1.04760663 | 0.024670274 | 1.121925882 | 1.053542709 |
| PVRL2_LDL | rs7254892 | -4.233415936 | -4.372795008 | -4.372804401 | -4.001612523 | 0.019426526 | 0.019429144 | 0.053732822 |
| PVRL2_LDL | rs6857 | 2.954739539 | 2.746756552 | 2.746751712 | 2.578484194 | 0.043256923 | 0.043258936 | 0.141568085 |
| LDLR_LDL | rs2228671 | -2.536806016 | -2.080417869 | -2.084374614 | -2.465821867 | 0.208290141 | 0.204694174 | 0.005038749 |
| LDLR_LDL | rs5742911 | 0.284671428 | -0.264873243 | -0.244254179 | -0.089699384 | 0.301999346 | 0.279762298 | 0.140153505 |
| SMARCA4_LDL | rs3786725 | -0.458480358 | -0.502952894 | -0.502942099 | -0.561125397 | 0.001977807 | 0.001976846 | 0.010536004 |
| SMARCA4_LDL | rs11668477 | -0.384488201 | -0.605748151 | -0.605762386 | -0.483114026 | 0.048955966 | 0.048962265 | 0.009727053 |
| SMARCA4_LDL | rs17248720 | -0.505704778 | -0.45214849 | -0.452133054 | -0.687058297 | 0.002868276 | 0.00286993 | 0.032889099 |
| CELSR2_LDL | rs7528419 | -2.367292734 | -2.369827122 | -2.369811977 | -2.474215449 | 6.42312E-06 | 6.34659E-06 | 0.011432467 |
| CELSR2_LDL | rs11577931 | -0.790352261 | -0.699528801 | -0.6995259 | -1.030272049 | 0.008248901 | 0.008249428 | 0.057561505 |
| APOB_LDL | rs41288783 | 1.76947297 | 3.420005171 | 3.419999507 | 1.04556602 | 2.724256546 | 2.724237849 | 0.524041273 |
| APOB_LDL | rs1367117 | 2.910185185 | 2.25713605 | 2.257048196 | 2.758643823 | 0.426473173 | 0.426587926 | 0.022964784 |
| APOB_LDL | rs17398765 | 1.622196991 | 1.643590202 | 1.643585463 | 2.065937434 | 0.000457669 | 0.000457467 | 0.196905581 |
| APOB_LDL | rs1713222 | -3.116669924 | -3.351087817 | -3.351115573 | -3.111946112 | 0.054951749 | 0.054964762 | 2.23144E-05 |
| NOS1AP_QTc | rs10429888 | 2.490911309 | 2.377472234 | 2.471243247 | 2.465473043 | 0.012868424 | 0.000386833 | 0.000647105 |
| NOS1AP_QTc | rs12143842 | 1.190883833 | 1.104416056 | 1.11761382 | 1.391537871 | 0.007476676 | 0.005368495 | 0.040262043 |
| NOS1AP_QTc | rs16857031 | 1.533415746 | 1.857592491 | 1.753544599 | 1.879719059 | 0.105090562 | 0.048456712 | 0.119925984 |
| NOS1AP_QTc | rs4657166 | 1.169541099 | 1.043227506 | 1.242416028 | 1.235815155 | 0.015955124 | 0.005310755 | 0.00439225 |
| NOS1AP_QTc | rs10918859 | 2.843777122 | 2.867508711 | 2.692102924 | 3.105290835 | 0.000563188 | 0.023005062 | 0.068389422 |
| SCN5A10A_PR | rs62242769 | -2.993566176 | -3.009937739 | -3.240790044 | -3.251602378 | 0.000268028 | 0.061119641 | 0.066582681 |
| SCN5A10A_PR | rs55824920 | 2.500806266 | 2.604599146 | 2.393384496 | 2.336577518 | 0.010772962 | 0.011539437 | 0.026971082 |
| SCN5A10A_PR | rs6801957 | 3.716071862 | 3.69823516 | 3.812035923 | 3.733048072 | 0.000318148 | 0.009209101 | 0.000288192 |
| LDLR_TC | rs6511720 | 1.687022604 | 1.447187453 | 1.915661114 | 1.807973152 | 0.0575209 | 0.052275568 | 0.014629035 |
| LDLR_TC | rs6511721 | -0.602185521 | -0.128842372 | -0.31357412 | -0.445675498 | 0.224053736 | 0.08329654 | 0.024495387 |
| LDLR_TC | rs73015030 | 0.207349996 | -0.289817765 | 0.10404387 | 0.268886705 | 0.247175783 | 0.010672156 | 0.003786766 |
| LDLR_TC | rs2228671 | -2.454204799 | -1.988458782 | -2.55580715 | -2.459819125 | 0.216919353 | 0.010323038 | 3.15206E-05 |
| LDLR_TC | rs2738447 | -0.561471057 | 0.090319352 | -0.069558501 | -0.172280345 | 0.424830737 | 0.241977963 | 0.15146941 |
| LDLR_TC | rs5742911 | 0.183467755 | -0.476513532 | -0.446809255 | -0.316276849 | 0.4355753 | 0.397249109 | 0.24974467 |
| SMARCA4_TC | rs8099996 | -0.518252473 | -0.440740353 | -0.403389012 | -0.370401741 | 0.006008129 | 0.013193615 | 0.021859839 |
| SMARCA4_TC | rs8102273 | -0.42604202 | -0.518089078 | -0.576575704 | -0.620867796 | 0.008472661 | 0.02266039 | 0.037957083 |
| SMARCA4_TC | rs73015007 | 0.995280545 | 0.950646218 | 0.973119443 | 1.083393054 | 0.001992223 | 0.000491114 | 0.007763814 |
| SMARCA4_TC | rs11668477 | 0.522288168 | 0.489515779 | 0.542662416 | 0.607962843 | 0.001074029 | 0.00041511 | 0.00734015 |
| SMARCA4_TC | rs17248720 | -1.112347693 | -1.006888526 | -1.024401803 | -1.222934241 | 0.011121636 | 0.007734479 | 0.012229385 |
| PVRL2_TC | rs7254892 | -2.69671704 | -2.782619317 | -2.782623456 | -2.565549121 | 0.007379201 | 0.007379912 | 0.017205023 |
| PVRL2_TC | rs6857 | 2.230858694 | 2.090220512 | 2.090217346 | 1.998877487 | 0.019779098 | 0.019779989 | 0.05381528 |
| APOB_TC | rs1367117 | 2.285083105 | 2.213048573 | 2.212774475 | 2.430656431 | 0.005188974 | 0.005228538 | 0.021191593 |
| APOB_TC | rs1800481 | -3.207854237 | -3.503628698 | -3.50372835 | -3.195454873 | 0.087482532 | 0.087541491 | 0.000153744 |
| APOB_TC | rs17398765 | 1.830515038 | 1.616441712 | 1.616408118 | 2.09581929 | 0.045827389 | 0.045841773 | 0.070386346 |
| Mean Square Error | | 0.067894005 | 0.069765621 | 0.062671877 | 0.062248606 | 0.184440754 | 0.192047818 | 0.292837007 |
| HAPRAP (SHAPEIT) accuracy improvement (%) | | | | | | | 0.760706332 | 10.83962522 |

Notation: traits refer to the region associated with a certain trait. SNP is the rs ID of SNPs. T_Mreg, T_HS, T_HP and T_GCTA are the t-statistics of multiple regression HAPRAP (SHAPEIT), HAPRAP (PLINK) and GCTA-COJO respectively. R_HS R_HP and R_G refer to the residuals of t-statistics of HAPRAP using haplotypes phased by SHAPEIT, the residuals of HAPRAP using haplotypes phased by PLINK and the residual of GCTA-COJO respectively. Mean Square Error is the residuals of sum of squares divided by the number of degree of freedom. HAPRAP (SHAPEIT) accuracy improvement is the percentage of accuracy improvement of HAPRAP using SHAPEIT compare to HAPRAP using PLINK and GCTA-COJO.

**Table S11.** The concordance correlation coefficient analysis.

| Methods | Cohorts | CCC | 95% CI of CCC | C.b |
| --- | --- | --- | --- | --- |
| HAPRAP (SHAPEIT) vs Mreg | BWHHS | 0.998 | 0.997~0.998 | 0.999 |
| GCTA-COJO vs Mreg | BWHHS | 0.997 | 0.997~0.998 | 0.999 |

Notations: CCC represents the centre estimate of concordance correlation coefficient and 95% CI of CCC is the 95% confidence interval of the centre estimate. Closer the CCC estimates the better agreement of two methods. C.b refers to the bias correction factor, which is a measurement of how far the best-fit line deviates from a line at 45 degrees. No deviation from the 45 degree line occurs when C.b = 1.

**Table S12.** HAPRAP and GCTA-COJO joint effect analysis of identifying two independent effect SNPs associated with gallbladder disease in *ABCG 5* and *ABCG8* regions.

| SNP |  | HAPRAP | | |  | GCTA-COJO | | |
| --- | --- | --- | --- | --- | --- | --- | --- | --- |
|  |  | beta | SE | p value |  | beta | SE | p value |
| rs4299376 |  | -0.223 | 0.033 | 8.86x10^-12^ |  | -0.221 | 0.033 | 1.92x10^-11^ |
| rs4953023 |  | 0.688 | 0.054 | 5.87x10^-38^ |  | 0.712 | 0.053 | 5.50x10^-41^ |

**Table S13.** Summary of 3 multiple associated SNPs at *NOS1AP* for QTc interval.

| SNP | Chr. | Location (bp) | Nearest gene | Allele | MAF | UCLEB meta-analysis | | |  | HAPRAP, haplotypes from BWHHS | | |
| --- | --- | --- | --- | --- | --- | --- | --- | --- | --- | --- | --- | --- |
|  |  |  |  |  |  | beta | Std Err | P |  | BETA | Std Err | P |
| rs10429888 | 1 | 162024987 | *NOS1AP* | A/G | 0.1291 | 4.0402 | 0.6642 | 1.18x10^-9^ |  | 3.707087735 | 0.642 | 8.07x10^-9^ |
| rs16857031 | 1 | 162112910 | *NOS1AP* | G/C | 0.1469 | 2.8277 | 0.6276 | 6.61x10^-6^ |  | 2.838606397 | 0.6082 | 3.104x10^-6^ |
| rs10918859 | 1 | 162169268 | *NOS1AP* | A/G | 0.1784 | 3.5747 | 0.5825 | 8.41x10^-10^ |  | 2.599107315 | 0.5553 | 2.913x10^-6^ |

The UCLEB meta-analysis betas are the marginal SNPs effects of the SNPs. For the HAPRAP analysis, UCLEB meta-analysis summary statistics and individual-level haplotypes from 1980 BWHHS individuals were used. The HAPRAP betas are the independent SNP effects of the SNPs. Chr represents the chromosomes of the SNPs. MAF represent the minor allele frequencies of the SNPs. The Standard errors are noted as Std Err and the P values are noted as P. The GCTA-COJO results was not presented in this table since only top hit rs12143842 was detected as independent signal.

**Table S14**. Overview of Strongly Supported Functional SNPs in Linkage Disequilibrium with Multiple Associated Lead SNPs.

| Lead SNP | Lead SNP score | Best SNP in LD | score | Distance to lead SNP (bp) | r2 with lead SNP |
| --- | --- | --- | --- | --- | --- |
| rs10429888 | 2b | - | - | - | 1 |
| rs10918859 | 5 | rs4656362 | 2b | 49833 | 0.71 |
| rs16857031 | 5 | rs1337072 | 1d | 8252 | 0.748 |

The score for the lead SNP (and score for the highest-scoring SNP in LD with the lead SNP) is the RegulomeDB (Boyle AP et al, 2012) results for that SNP. Each functional SNP in this table overlaps TF bindings, motifs, DNaseI foot prints and DNaseI peaks.

**Table S15.** Comparison of haplotype phasing accuracy in the simulated data

| Haplotypes | Haplotype frequencies | PLINK | SHAPEIT | Error_SHAPEIT | Error_PLINK |
| --- | --- | --- | --- | --- | --- |
| 212 | 0.000993 | 0.001017 | 0.04103 | 0.040037 | 2.4E-05 |
| 121 | 0.000993 | 0.001037 | 0.06684 | 0.065847 | 0.000044 |
| 112 | 0.0501428 | 0.05067 | 0.07697 | 0.0268272 | 0.0005272 |
| 122 | 0.0588642 | 0.05867 | 0.07783 | 0.0189658 | 0.0001942 |
| 211 | 0.0788642 | 0.07872 | 0.11369 | 0.0348258 | 0.0001442 |
| 221 | 0.1301428 | 0.1301 | 0.12132 | 0.0088228 | 4.28E-05 |
| 222 | 0.19 | 0.189 | 0.12281 | 0.06719 | 0.001 |
| 111 | 0.49 | 0.4908 | 0.37951 | 0.11049 | 0.0008 |
| Total difference | | | | 0.3730056 | 0.0027764 |

Notation: haplotypes refer to the 8 haplotypes of the 3 SNPs model. Haplotype frequencies are the default frequencies of the 8 haplotypes we set. PLINK refers to the haplotype frequencies estimated by PLINK. SHAPEIT refers to the haplotype frequencies estimated by SHAPEIT. Error_SHAPEIT and Error_PLINK are the difference between our default haplotype frequencies and that estimated by SHAPEIT and PLINK respectively. For SHAPEIT, we set 1500 as the number of conditional states, which is 15 times higher than the default states.

In this comparison, we simulated unphased genotypes for 100,000 individuals using the haplotypes and haplotype frequencies we set (Column 1 and 2 in the above table). We phased the genotypes using both PLINK and SHAPEIT. PLINK works much better in this case (error). We think there are several reason. Firstly, PLINK uses E-M algorithm to phase haplotypes, which works well in model with less than 10 SNPs (in this case, there are three SNPs in the model). Secondly, since the population is simulated, we cannot use the genetic map file for SHAPEIT, which affected the accuracy of SHAPEIT. Thridly, SHAPEIT need a large region to achieve its best performance (say 500 SNPs). Three SNPs model is far too small. So for this simulation study, we decided to use PLINK to phase haplotypes.

**Table S16.** Meta-Analysis Summary Statistics for the SNPs Significantly Associated with Gallbladder Disease in ABCG 5 / 8 Region.

| SNP | A1 | A2 | MAF | b | se | p | N |
| --- | --- | --- | --- | --- | --- | --- | --- |
| rs6720173 | C | G | 0.1583 | 0.2561 | 0.0386 | 3.23E-11 | 15213 |
| rs10208987 | C | A | 0.0799 | 0.3782 | 0.0502 | 4.77E-14 | 15213 |
| rs4148189 | A | G | 0.1139 | 0.2538 | 0.0441 | 8.66E-09 | 15213 |
| rs4245786 | G | A | 0.2425 | 0.1423 | 0.0333 | 1.88E-05 | 15213 |
| rs4299376 | C | A | 0.3154 | -0.2829 | 0.0325 | 3.10E-18 | 15213 |
| rs4953023 | A | G | 0.0658 | 0.7625 | 0.0523 | 3.57E-48 | 15213 |
