# Supplementary material for: HAPRAP: a haplotype-based iterative method for statistical fine mapping using GWAS summary statistics
Source: Bioinformatics. 2016 Sep 1;33(1):79–86. doi: 10.1093/bioinformatics/btw565 (PMC5544112; doi:10.1093/bioinformatics/btw565)
Supplement: Supplementary Data [file btw565_supp.zip › btw565_Supp2.docx]

**Figure S1. Population Simulation in the SimHAPRAP Procedure.**

**
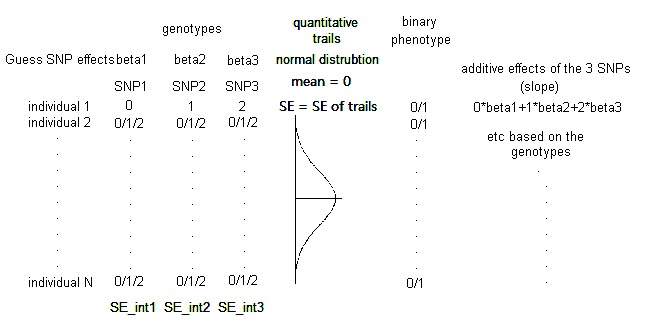
**

**Figure S2. Performance Comparison of HAPRAP, GCTA and Multiple Regression Using Artificial Meta-Analyses of the Simulated Populations.**


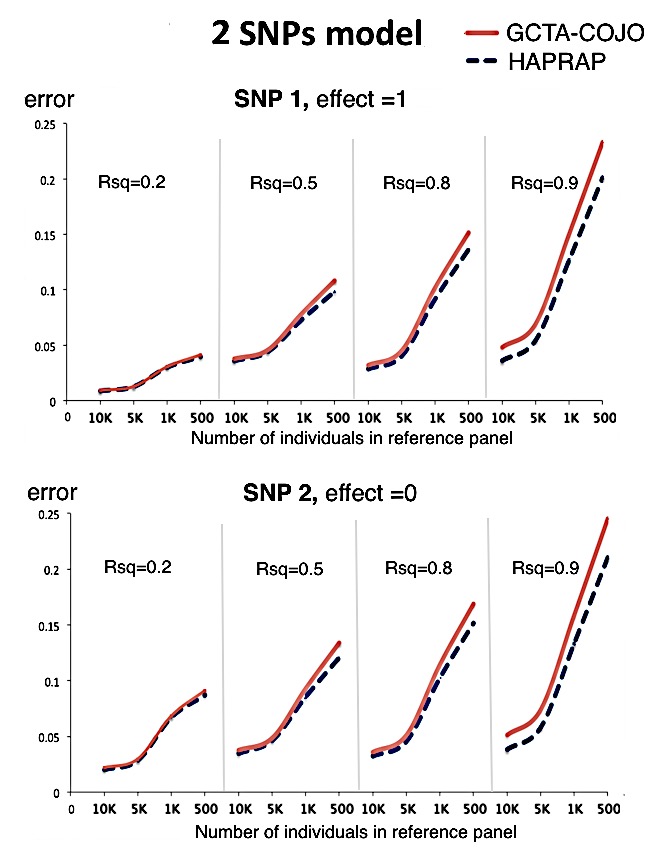
A

B


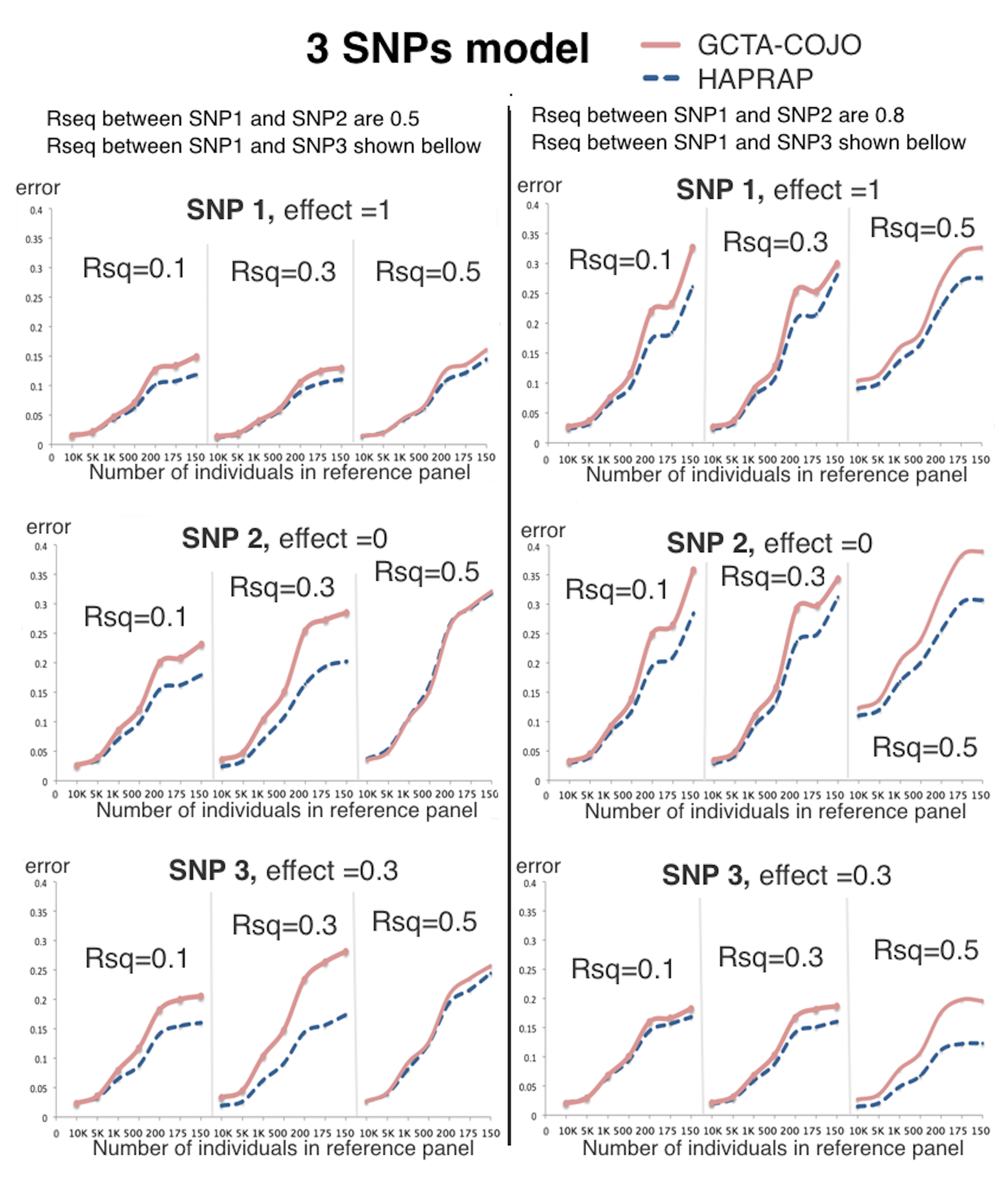


C


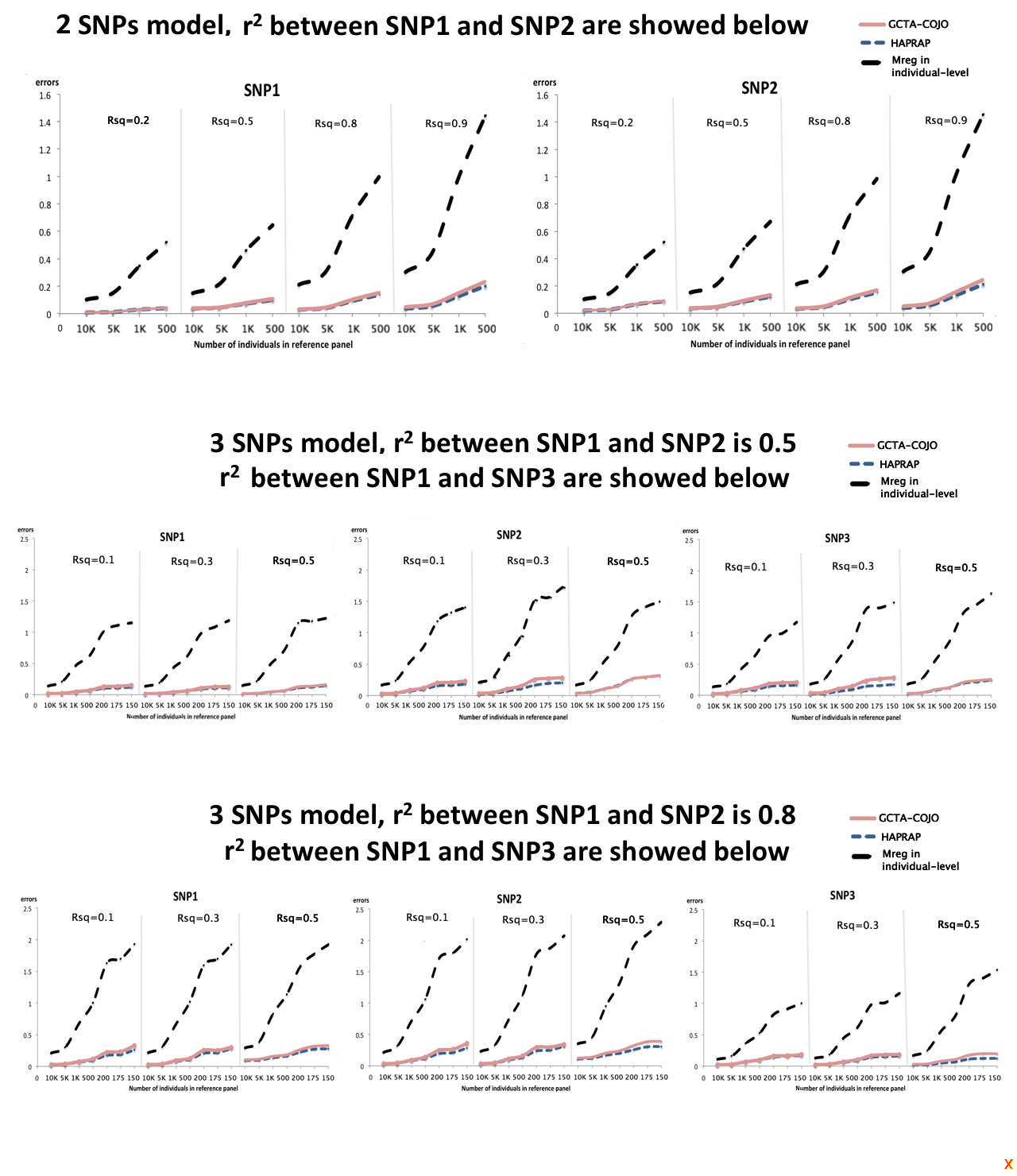


Error (represented as a bar) is defined as the difference between the gold standard and the mean (with 95% CI) of 1000 replications. N is the number of individuals in the reference panel. **A,** the comparison between HAPRAP and GCTA for the 2-SNPs models with r^2^=0.8. **B**, the comparison between HAPRAP and GCTA for the 3-SNPs models with r^2^=0.5. X-axis is the number of individuals in the reference panel in Log scale..**C,** the comparison of multiple regression, HAPRAP and GCTA in the 3-SNPs models.

**Figure S3. The Multidimensional Scaling Plot of 1000 Genome CEU and GBP Population Together with BWHHS Individuals.**


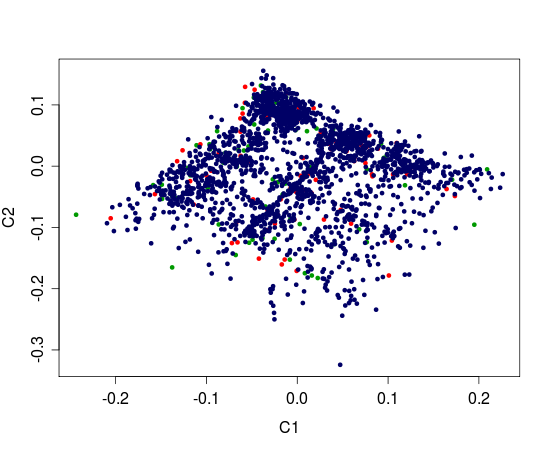


Points in green are from the CEU population, points in red are from the GBR population and points in blue are from BWHHS. The three populations cluster together which suggests no population stratification between the populations.

**Figure S4. Histogram of T-statistics of joint SNP effects of multiple regression, HAPRAP and GCTA_COJO using BWHHS or 1000 Genome individual-level data.**


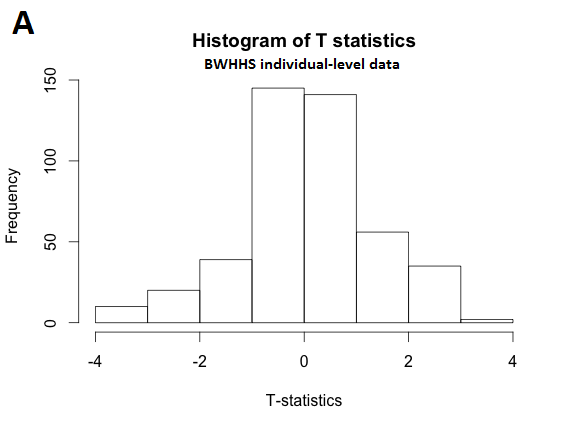


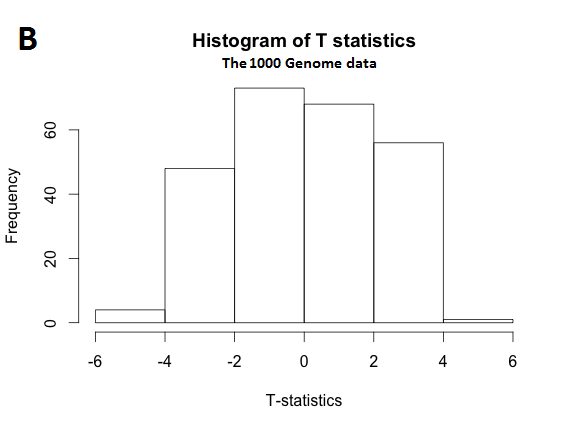


**A.** Histogram of the T-statistics of joint SNP effects of multiple regression, HAPRAP (phased by SHAPEIT), HAPRAP (phased by PLINK) and GCTA-COJO using BWHHS individual-level data. **B.** Histogram of the T-statistics of joint SNP effects of the four methods using the 1000 Genome data.

**Figure S6. Performance Comparison of HAPRAP and GCTA-COJO Partial Effect Analysis Using BWHHS Unphased Genotypes.**


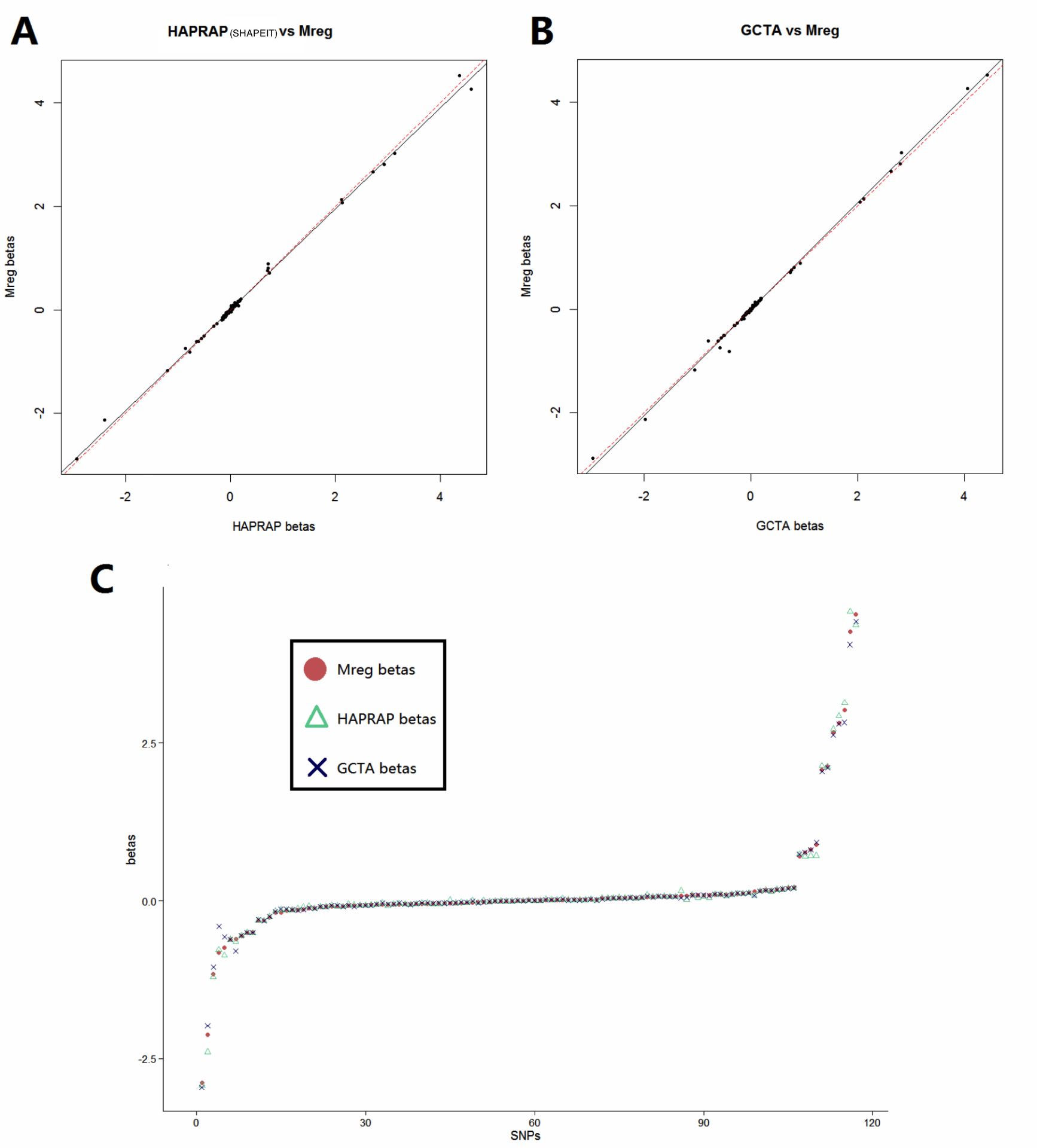


(A) Scatter plot of multiple regression betas vs HAPRAP betas (using SHAPEIT). (B) Scatter plot of multiple regression betas vs GCTA-COJO betas. In (A) and (B), the light color is the Y=X line, the black line is the best-fit line. (C) Pair plot of Multiple regression betas, HAPRAP betas and Multiple regression betas. SNPs were sorted by multiple regression betas in ascending order. The full circles represent the multiple regression betas; clear triangles represent the HAPRAP betas and cross represent the GCTA-COJO betas. Detailed results were listed in Table S1.

**
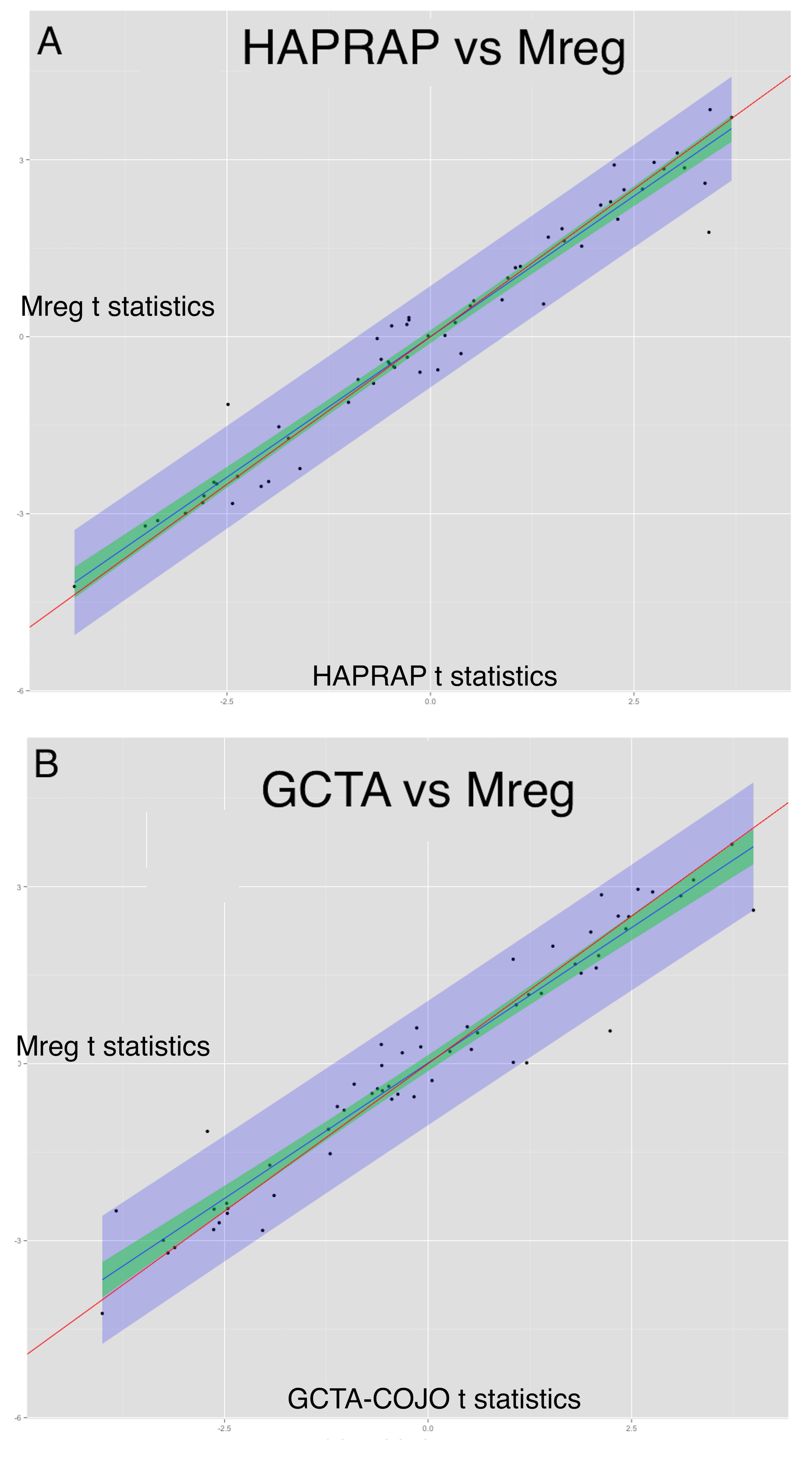
Figure S7. Performance Comparison between HAPRAP and GCTA-COJO Using the 1000 Genomes Phased Genotypes.**


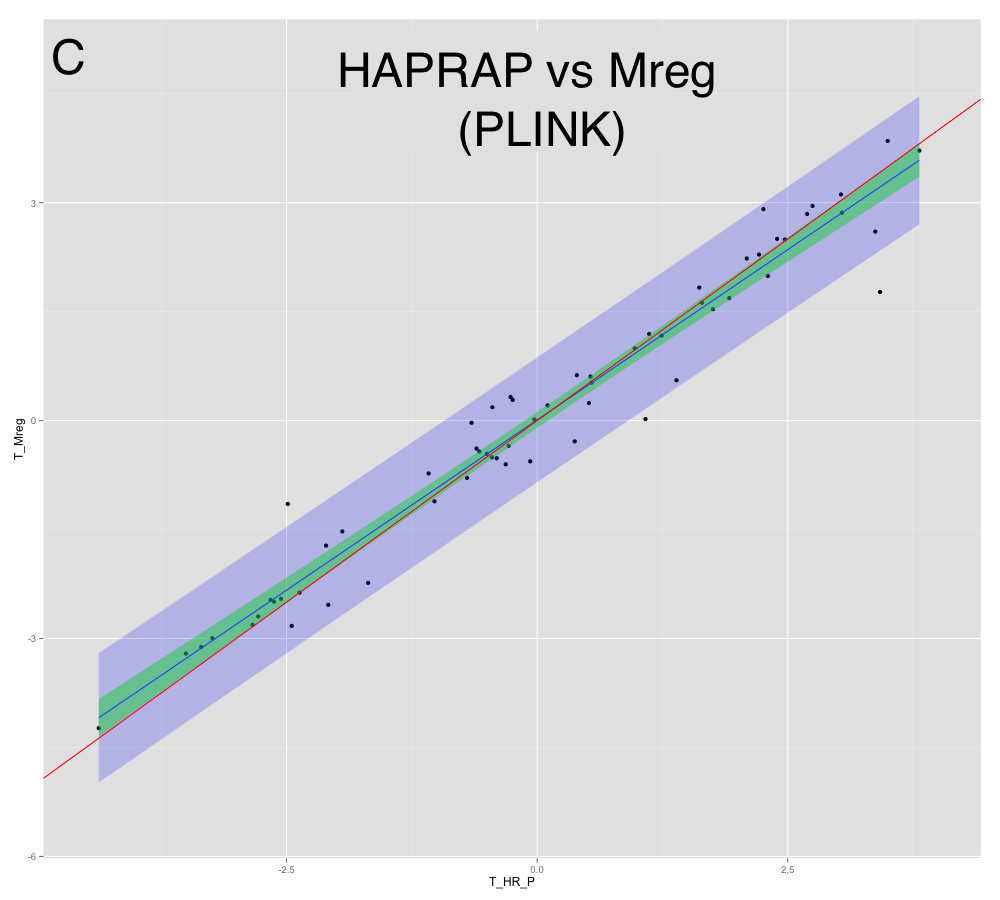


(A) Scatter plot of Mreg t-statistics vs HAPRAP t-statistics. (B) Scatter plot of Mreg t-statistics vs. GCTA-COJO t-statistics. (C) Scatter plot of Mreg t-statistics vs HAPRAP (PLINK) t-statistics.The longer light line is the Y=X line, the shorter line is the line of best fit. Inner zone and outer zone are the 95% confidence interval and prediction interval of the line of best fit respectively. Detailed results were listed in Table S2.
