# Supplementary material for: HAPRAP: a haplotype-based iterative method for statistical fine mapping using GWAS summary statistics
Source: Bioinformatics. 2016 Sep 1;33(1):79–86. doi: 10.1093/bioinformatics/btw565 (PMC5544112; doi:10.1093/bioinformatics/btw565)
Supplement: Supplementary Data [file btw565_supp.zip › btw565_Supp3.docx]

## Text S1: The UCLEB Consortium

T.S. (Department of Epidemiology and Public Health, UCL Institute of Epidemiology and Health Care, University College London, 1-19 Torrington Place, London WC1E 6BT, UK); J.E. (Department of Epidemiology and Public Health, UCL Institute of Epidemiology and Health Care, University College London, 1-19 Torrington Place, London WC1E 6BT, UK); C.E.D. (Department of Non-Communicable Disease Epidemiology, London School of Hygiene and Tropical Medicine, London WC1E 7HT, UK); S.S. (University College London Genetics Institute, Department of Genetics, Environment and Evolution, Gower St, London WC1E 6BT, UK); J.W. (University College London Genetics Institute, Department of Genetics, Environment and Evolution, Gower St, London WC1E 6BT, UK); C.G. (University College London Genetics Institute, Department of Genetics, Environment and Evolution, Gower St, London WC1E 6BT, UK); S.M.L.(Centre for Population Health Sciences, University of Edinburgh, Teviot Place, Edinburgh EH8 9AG, UK); D.Z. (University College London Genetics Institute, Department of Genetics, Environment and Evolution, Gower St, London WC1E 6BT, UK); Alana Cavadino (MRC Centre of Epidemiology for Child Health, Department of Population Health Sciences, UCL; Institute of Child Health, University College London, 30 Guilford Street, London WC1N 1EH, UK); C.F.(Department of Epidemiology and Public Health, UCL Institute of Epidemiology and Health Care, University College London, 1-19 Torrington Place, London WC1E 6BT, UK); Andrew Wong (MRC Unit for Lifelong Health and Ageing, 33 Bedford Place, London WC1B 5JU, UK); Antoinette Amuzu (Department of Non-Communicable Disease Epidemiology, London School of Hygiene and Tropical Medicine, London WC1E 7HT, UK); Ken Ong (MRC Unit for Lifelong Health and Ageing, 33 Bedford Place, London WC1B 5JU, UK; MRC Epidemiology Unit, Institute of Metabolic Science, Addenbrooke’s Hospital, Box 285, Cambridge CB2 0QQ, UK); T.R.G. (MRC Integrative Epidemiology Unit, School of Social and Community Medicine, University of Bristol, Oakfield House, Oakfield Grove, Bristol BS8 2BN, UK); M.V.H. (Department of Epidemiology and Public Health, UCL Institute of Epidemiology and Health Care, University College London, 1-19 Torrington Place, London WC1E 6BT, UK); Helen Warren (Department of Non-Communicable Disease Epidemiology, London School of Hygiene and Tropical Medicine, London WC1E 7HT, UK); D.I.S. (Department of Epidemiology and Public Health, UCL Institute of Epidemiology and Health Care, University College London, 1-19 Torrington Place, London WC1E 6BT, UK); Teri-Louise Davies (MRC Centre for Causal Analyses in Translational Epidemiology, School of Social and Community; Medicine, University of Bristol, Oakfield House, Oakfield Grove, Bristol BS8 2BN, UK); F.D.(Centre for Cardiovascular Genetics, Department of Medicine, British Heart Foundation Laboratories, Rayne Building, Royal Free and University College Medical School, 5 University Street, London, WC1E 6JF, UK); J.C. (Centrefor Cardiovascular Genetics, Department of Medicine, British Heart Foundation Laboratories, Rayne Building, Royal Free and University College Medical School, 5 University Street, London, WC1E 6JF, UK); R.S. (Centre for Clinical Pharmacology, University College London, London WC1E 6JF, UK), M.C. (William Harvey Research Institute, Barts and the London Queen Mary’s School of Medicine and Dentistry, John Vane Building, Charterhouse Square, London EC1M 6BQ, UK); Shah Ebrahim (Department of Non-Communicable Disease Epidemiology, London

School of Hygiene and Tropical Medicine, London WC1E 7HT, UK); D.A.L. (MRC Centre for Causal Analyses in Translational Epidemiology, School of Social and Community; Medicine, University of Bristol, Oakfield House, Oakfield Grove, Bristol BS8 2BN, UK); P.J.T. (Centre for Cardiovascular Genetics, Department of Medicine, British Heart Foundation Laboratories, Rayne Building, Royal Free and University College Medical School, 5 University Street, London, WC1E 6JF, UK); S.E.H. (Centre for Cardiovascular Genetics, Department of Medicine, British Heart Foundation Laboratories, Rayne Building, Royal Free and University College Medical School, 5 University Street, London, WC1E 6JF, UK); Christine Power (MRCCentre of Epidemiology for Child Health, Department of Population Health Sciences, UCL; Institute of Child Health, University College London, 30 Guilford Street, London WC1N 1EH, UK); Elina Hypponen (MRC Centre of Epidemiology for Child Health, Department of Population Health Sciences, UCL; Institute of Child Health, University

College London, 30 Guilford Street, London WC1N 1EH, UK); R.M. (MRC Unit for Lifelong Health and Ageing, 33 Bedford Place, London WC1B 5JU, UK); Rebecca Hardy (MRC Unit for Lifelong Health and Ageing, 33 Bedford Place, London WC1B 5JU, UK); Diana Kuh (MRC Unit for Lifelong Health and Ageing, 33 Bedford Place, London WC1B 5JU, UK); Nicholas Wareham (MRC Epidemiology Unit, Institute of Metabolic Science, Addenbrooke’s Hospital, Box 285, Cambridge CB2 0QQ, UK); Claudia Langenberg (MRC Epidemiology Unit, Institute of Metabolic Science, Addenbrooke’s Hospital, Box 285, Cambridge CB2 0QQ, UK; Department of Epidemiology and Public Health, UCL Institute of Epidemiology and Health Care, University College London, 1-19 Torrington Place, London WC1E 6BT, UK); Yoav BenShlomo (School of Social and Community Medicine, University of Bristol, Canynge Hall, 39 Whatley Road, Bristol BS8 2PS, UK); I.N.D. (MRC Centre for Causal Analyses in Translational Epidemiology, School of Social and Community; Medicine, University of Bristol, Oakfield House, Oakfield Grove, Bristol BS8 2BN, UK); Peter Whincup (Division of Population Health Sciences and Education, St George’s, University of London, Cranmer

Terrace, London SW17 0RE, UK); R.M. (Department of Primary Care and Population Health, University College London, Royal Free Campus, Rowland Hill Street, London NW3 2PF, UK); J.P. (Centre for Population Health Sciences, University of Edinburgh, Teviot Place, Edinburgh EH8 9AG, UK); ME.K. (Department of Epidemiology and Public Health, UCL Institute of Epidemiology and Health Care, University College London, 1-19 Torrington Place, London WC1E 6BT, UK); MI.K. (Department of Epidemiology and Public Health, UCL Institute of Epidemiology and Health Care, University College London, 1-19 Torrington Place, London WC1E 6BT, UK); V.P. (University College London Genetics Institute, Department of Genetics, Environment and Evolution, Gower St, London WC1E 6BT, UK); F. D. (Department of Non-Communicable Disease Epidemiology, London School of Hygiene and Tropical Medicine, London WC1E 7HT, UK);

J.C.W. (Genetics Division, Research and Development, GlaxoSmithKline, NFSP, Harlow CM19 5AW, UK); J.P.C. (Department of Epidemiology and Public Health; UCL Institute of Epidemiology and Health

Blood lipids and CHD Page 11 of 13 at University Library on February 28, 2014 http://eurheartj.oxfordjournals.org/ Downloaded from Care, University College London, 1-19 Torrington Place, London WC1E 6BT, UK; Department of Non-Communicable Disease Epidemiology, London School of Hygiene and Tropical Medicine, London WC1E 7HT, UK); A.D.H. (Department of Epidemiology and

Public Health, UCL Institute of Epidemiology and Health Care, University College London, 1-19 Torrington Place, London WC1E 6BT, UK; Centre for Clinical Pharmacology, University College London, London

WC1E 6JF, UK).

## Text S2. Simulation for equation (2)

Given a 3x8 haplotype matrix of 3 SNPs and 8 haplotypes and a set of true partial SNP effects, we estimated the marginal SNP effects by using equation (2). Using the same haplotype matrix and partial SNP effects, we simulated genotypes and phenotypes of 1000 individuals, and estimated the marginal SNP effect of these 3 SNPs using a simple single regression model. We found that these two approaches always returned the same marginal SNP effects

## Text S3: Sample dataset

*ALSPAC individual-level haplotypes*

A total of 18420 subjects were genotyped using the Illumina HumanHap550 quad genome-wide SNP genotyping platform (Illumina Inc., San Diego, CA, USA) by 23andMe subcontracting the Wellcome Trust Sanger Institute, Cambridge, UK and the Laboratory Corporation of America (LabCorp Holdings., Burlington, NC, USA). PLINK software (v1.07) was used to carry out quality control measures. Individuals were excluded from further analysis on the basis of having incorrect gender assignments, minimal or excessive heterozygosity (0.320 and .0.345 for the Sanger data and ,0.310 and .0.330 for the LabCorp data), disproportionate levels of individual missingness (.3%), evidence of cryptic relatedness (.10% IBD) and being of non-European ancestry (as detected by a multidimensional scaling analysis seeded with HapMap 2 individuals). EIGENSTRAT analysis revealed no additional obvious population stratification and genome-wide analyses with other phenotypes indicate little genomic inflation). After quality control, 8263 unrelated children were selected as LD reference panel for all analysis. Genotypes were phased together using ShapeIt (version 2 revision 727). SNPs were filtered to be in Hardy-Weinberg equilibrium (P > 5×10-7).

*BWHHS individual-level dataset*

We used the individual-level data of the BWHHS cohort (Lawlor et al., 2003). Among all the BWHHS individuals, 1980 samples are genotyped by Illumina Cardio-MetaboChip (Voight et al. 2012) and 3445 individuals were genotyped using the Human cardiovascular disease (HumanCVD) BeadChip (Illumina, San Diego, CA). We removed SNPs with Hardy-Weinberg disequilibrium p value smaller than 0.001 from the analysis. In total, 113 SNPs were selected within genomic regions listed in Table S1. To test the collinearity of multiple SNPs within each of the above genomic regions, variance inflation factors (VIF) were estimated using R package 'car'. SNPs with VIF higher than 10 were removed for further analysis (Armitage and Berry 1994).

*The 1000 Genomes Project individual-level dataset*

We combined the 1000 Genomes Project data unrelated Utah Residents (CEPH) with Northern and Western European ancestry (CEU) individuals and British in England and Scotland (GBP) individuals (The 1000 Genomes Project Consortium et al., 2010). From the 113 selected SNPs (Table S1), we removed SNPs not included in the 1000 Genomes Project database and SNPs with high VIF. 61 SNPs remained for this comparison (Table S2). The Principal-Component Analysis (PCA) of the 61 SNPs showed no population stratification between the two populations (Figure S3).

*Simulations of meta-analyses*

We tested 2-SNPs models and 3-SNPs models in this simulation, and also varied the genetic architecture and LD structure of the simulated genotypes. We did not use larger haplotypes because no additional constraints on pair-wise disequilibrium values exist in a four-locus situation, except those that involve three-locus combinations containing the two loci of interest (Robinson et al., 1991). To mimic complex models with 2 or 3 SNPs, we firstly assumed that SNP1 is a top hit with independent effect of 1. SNP2 was set as negative control without any independent effect. We used it to test whether the method can eliminate the bystander SNP. Moreover, SNP3 was set as a secondary signal with a small independent effect of 0.3. We set this effect size to test whether HAPRAP can detect this small effect even if the secondary SNP (SNP3) is in high LD to the top hit (SNP1).

We simulated data to represent genetic architectures with common variants or rare variants. In the common variants setting, we set the MAFs as 0.4, 0.38 and 0.3 respectively for SNP 1, 2 and 3. In the rare variant setting, we set MAFs for SNP1 and SNP2 to be 0.093 and 0.083, respectively (Table S6). We simulated data under models with different LD correlations among SNPs (Table S4). For each model, we simulated unphased genotypes and phenotypes of 100,000 individuals using the same method described above in the simHAPRAP section. Then, single-SNP associations and MAFs were estimated using PLINK. To mimic a real world situation, we assumed that only the summary statistics (betas, standard errors and MAFs) and a reference genotype panel of size N were available, where N were 10,000, 5,000, 1,000, 500, 200, 175 or 150 individuals with genotypes randomly picked from the population pool. The haplotypes were phased using PLINK.

*Meta-analysis datasets*

We used meta-analysis summary statistics of a consortium analysis of gallbladder disease (GBD) (Rodriguez et al., 2014). The meta-analysis included 15213 individuals.

In addition, we used summary statistics of UCLEB meta-analysis on 7106 individuals for QTc interval. The UCLEB meta-analysis of QTc interval included three participating studies: BWHHS, British Regional Heart Study (BRHS) (Walker et al., 2004) and Whitehall II study (WHII) (Marmot and Brunner, 2005).

*GIANT height meta-analysis*

We selected the largest and most up to date human height meta-analysis from the GIANT consortium (Wood et al., 2014). In total 253288 indviduals were included in this meta-analysis. We selected three genomic regions, *ACAN*, *ADAMTS17* and *PTCH1* for our case study. These regions were reported to contain multiple association signals with human height. The summary level data was downloaded from the GIANT consortium webpage (<https://www.broadinstitute.org/collaboration/giant/index.php/GIANT_consortium_data_files>). We used all SNPs within these three regions (782 SNPs for *ACAN*, 1477 SNPs for *ADAMTS17* and *1936* SNPs for *PTCH1*). We also simulated a meta-analysis using data from the *ACAN* region. In total 23 SNPs were selected for this simulation.

## Text S4: Performance comparisons between HAPRAP and GCTA-COJO using BWHHS individual-level data and 1000 Genome project data

We tested HAPRAP using empirical genetic association data, the BWHHS individual-level data, and compared its performance against GCTA-COJO and multiple regressions. We first compared the performance of haplotype phasing methods (details in Text S5). Then we applied HAPRAP and GCTA-COJO using the summary statistics and individual-level genotypes of all BWHHS individuals. Multiple regression partial SNP effects were estimated by using the BWHHS individual-level genotypes and phenotypes, and set as gold standard. For each of the 113 SNPs, we calculated the t-statistics of partial SNP effects from multiple regression, HAPRAP and GCTA. Histograms of the t-statistics were drawn. Scatter plots were created to compare the HAPRAP t-statistics (and GCTA-COJO t-statistics) against multiple regression t-statistics. In addition, we calculated the concordance correlation coefficients (noted as CCC) between HAPRAP betas (or GCTA-COJO betas) and multiple regression partial SNP effects using the R package “eipR”. CCC measures the agreement between results from two methods. It combines measures of both precision and accuracy to determine how far the betas of different methods deviate from the line of perfect concordance (the y=x line) (Lin, 1989). The method (HAPRAP or GCTA-COJO) with CCC estimates closer to 1 got more agreement to the gold standard. In addition, pair plots were drawn between each pair of betas to compare the agreement between HAPRAP (GCTA-COJO) and multiple regression using R package “ggplot2”.

More importantly, we compared the performance of HAPRAP against GCTA-COJO using the 1000 Genomes Project European population (CEU+GBR) as a reference panel. HAPRAP was applied using haplotype data, whereas GCTA-COJO was run using genotype data. To quantify the performance of each method, we estimated the mean square error between multiple regression t-statistics and those of the other 3 methods. Scatter plot with 95% confidence interval and prediction interval were drawn for HAPRAP and GCTA-COJO.

We utilised individual-level data of ~2000 BWHHS individuals on a total of 115 SNPs to compare the accuracy of HAPRAP (haplotypes phased by both SHAPEIT and PLINK) and GCTA-COJO using multiple regression as gold standard (Table S1). As illustrated in Figure S4, the distribution of the t-statistics of partial SNP effects of the four methods are approximately normally distributed with a mean near zero, which fits the assumption of analysis of variance (ANOVA). We then used a two-way ANOVA to test the performance differences between the four methods. As shown in Table S9, there are no statistically differences amongst the results of multiple regression, HAPRAP (using PLINK and SHAPEIT) and GCTA-COJO.

Moreover, as shown in Figure S6A and B, all the points of the scatter plots were close to the y=x line, which suggests good correlations among the three methods. As illustrated in Table S11, HAPRAP (using SHAPEIT) agreed slightly better with multiple regression than GCTA-COJO (CCC estimate 0.9982 for HAPRAP and 0.9978 for GCTA-COJO). For the pair of plots shown in Figure S6C, we found that most of the HAPRAP betas are very close to the multiple regression partial SNP effects (gold standards).

We also compared HAPRAP and GCTA-COJO using the 1000 Genomes Project individual-level genotypes as a reference panel (N=175). For the 61 SNPs we tested, HAPRAP agreed better with multiple regression than GCTA-COJO. The 95% confidence interval and prediction interval of the line of best fit of HAPRAP was much more narrow than that of GCTA-COJO (Figure S7 and Table S2). To quantify this difference, we estimated the mean square error of the three methods (deviation between points and Y=X line). As shown in Table S10, HAPRAP’s mean square error (phased by SHAPEIT) is 10.84% lower than that of GCTA-COJO. This comparison indicates that HAPRAP is more reliable than GCTA-COJO when the sample size of the reference panel is limited.

## Text S5: Case studies of GBD and QTc interval

**Case study 1: meta-analysis of GBD**

HAPRAP is applicable to case-control studies. We demonstrated this by performing a case study in the *ABCG5* and *ABCG8* region, which was previously reported to contain more than one SNP robustly associated with gallbladder disease (GBD) (Buch et al., 2007). The summary results of 15213 individuals from a GBD meta-analysis and the individual-level genotypes of 3078 BWHHS individuals were used here. We applied HAPRAP and COJO to a total of 55 genotyped SNPs in this region. For the 55 SNPs we tested, HAPRAP identified rs4299376 and rs4953023 as independent effect SNPs (Table S12), which generally replicated the findings in the previous meta-analysis (Rodriguez et al., 2015). This proved the suitability of HAPRAP in the case-control study.

**Case study 2: UCLEB meta-analysis of QTc interval**

We applied HAPRAP using summary statistics from the UCLEB meta-analysis of QTc interval on 7106 individuals. Genotypes of 1980 individuals from BWHHS were used as a reference panel (Buch et al., 2007). In this case study, we focused on the *NOS1AP* region, which has been previously reported to be robustly associated with QTc interval (Arking, et al., 2006; Pfeufer, et al., 2009; Newton-Cheh et al., 2009; Marroni et al., 2009). The BWHHS cohort was imputed using the 1000 Genomes CEU reference panel. We used the best guess genotypes of the imputed SNPs. In total 199 imputed SNPs in this region were selected in this analysis. For the 199 SNPs we tested, HAPRAP identified three independently associated SNPs: rs10429888, rs16857031 and rs10918859 (Table S13). As a comparison, GCTA-COJO only identified the top hit, rs12143842. Then we compared HAPRAP results to previous findings in the literature, the first independent SNP, rs10429888, is in strong LD with a previously associated SNP, rs12143842, (r2=0.684 in the BWHHS population). This SNP was indicated as the most significant SNP in two previous reports (Pfeufer, et al., 2009; Newton-Cheh et al., 2009). Meanwhile, the second independent SNP, rs10918859, is in moderate LD with two previously reported SNPs, rs4657178 (r2=0.455 in BWHHS) and rs12029454 (r2=0.187 in BWHHS). Moreover, conditional on rs12143842, both were reported to be secondary signals in separate studies (Pfeufer, et al., 2009 and Newton-Cheh et al., 2009). In addition, there is evidence of correlation between the third independent SNP, rs16057031, and two different previously reported SNPs rs10494366 (r2=0.172 in BWHHS) and rs288058 (r2=0.148 in BWHHS) (Arking, et al., 2006; Marroni et al., 2009).

## Text S6: Explanation of COJO’s functionality

COJO consists of two functions:

1. an approximate joint SNP effect analysis function, which provides an effective way to estimate approximate joint SNP effect using GWAS summary-level statistics and pair-wise LD information from a reference panel. When using this function, COJO estimated the approximate variance-covariance matrix using allele frequencies reported on meta-analysis (Yang et al., 2012). The minor deviation between the allele frequencies reported on the meta-analysis and the true allele frequencies may cause minor deviation of the approximate joint SNP effect COJO estimated, even when the LD was perfectly estimated. We tested this possibility using genotype data of 7000 ALSPAC children (individual-level genotypes is available). Using actual genotype flag (multiple regression) and without using it (the approximate joint SNP effect analysis) returned results with minor deviation.
2. A multiple regression analysis function (using the actual genotype flag “--cojo-actual-geno”) when the full individual-level genotype is available, which returns the actual joint SNP effect as outcome. We tested such function using an ‘upside down’ study design, in which the GWAS was conducted using 1000 individuals from the ALSPAC cohort, while 16000 ALSPAC individuals were used as a reference panel for LD. This design returns misleading results (e.g. more than 20 independent signals within a region). So we suggest using the actual genotype flag with caution.

For all the comparisons between HAPRAP and COJO, we always assume that individual-level data is not available and only use COJO’s main function.

## Text S7: Functional annotation for the multiple associated SNPs for QTc interval

For *NOS1AP* region, we further analysed variants with r^2^ >= 0.7 (1000 Genome CEU) to the lead SNPs (defined as the multiple associated SNPs for QTc interval) identified using SNAP (Johnson, A D et al, 2008). We divided these SNPs into coding and non-coding and annotated with the FATHMM web-based tool (<http://fathmm.biocompute.org.uk/>) and RegulomeDB web-based tool (<http://regulome.stanford.edu/>) respectively.

For the three jointly associated SNPs for QTc interval (define as lead SNP), we found 25 proxy SNPs with strong LD (r^2^ >= 0.7) to them. RegulomeDB was used to annotate the 28 SNPs since all of them are in non-coding regions. We listed the lead SNPs and their best SNP in LD based on RegulomeDB score in Supplementary Table 7. The first lead SNP rs10429888 got the highest score. The second lead SNP rs10918859 got a score of 5. But another SNP in strong LD, rs4656362, got a score of 2b. The third SNP rs16857031 also got a low score of 5. But another SNP in strong LD, rs1337072, got a very high score of 1d.

As shown in Supplementary Table 7, rs1337072 is previously associated with an eQTL which affected *Myelin protein zero MPZ* (Montgomery et al., 2010). This protein is a major structural component of the myelin sheath. Interestingly, *MPZ* is also expressed in heart tissue with protein abundance of 59.7 ppm (Kline KG et al, 2008). So it will be worthwhile to further analyse the potential mechanism of *MPZ* in relation to QT interval.

## Text S8: Performance Comparison of haplotype phasing methods using BWHHS data

Haplotypes and haplotype frequencies are the core observations for HAPRAP. So we phased the haplotypes of the BWHHS individuals using both PLINK and SHAPEIT. For PLINK, we used the function “--hap-freq” to phase haplotypes. For SHAPEIT, we selected a 2Mb window for each genomic region and phased the haplotypes. The haplotypes of the selected SNPs were used for further analysis. We run HAPRAP based on the haplotypes of both of them and called the results HAPRAP_P and HAPRAP_S respectively. We compared the results of these two HAPRAP settings to multiple regression results so that we can suggest better option for users.

We further compared how different haplotype phasing approaches affect the accuracy of HAPRAP (A and Figure S7). As shown in Table S10, the results of HAPRAP using haplotypes phased by SHAPEIT resulted in an average accuracy improvement of 0.76% per SNP compared to the results of HAPRAP when using haplotypes phased by PLINK.

## Reference:

Arking, D.E., et al. (2006). A common genetic variant in the NOS1 regulator NOS1AP modulates cardiac repolarization. Nat. Genet. 38, 644–651.

Buch, S., et al. (2007). A genome-wide association scan identifies the hepatic cholesterol transporter ABCG8 as a susceptibility factor for human gallstone disease. Nat. Genet. 39, 995–999.

Marroni, F., et al. (2009). A genome-wide association scan of RR and QT interval duration in 3 European genetically isolated populations: the EUROSPAN project. Circ. Cardiovasc. Genet. 2, 322–328.

Pfeufer, A., et al. (2009). Common variants at ten loci modulate the QT interval duration in the QTSCD Study. Nat. Genet. 41, 407–414.

Teslovich, T.M., et al. (2010). Biological, clinical and population relevance of 95 loci for blood lipids. Nature 466, 707–713.

Armitage, P., and Berry, G. (1994). Statistical methods in medical research (Blackwell).

Lawlor, D.A., et al. (2003). Geographical variation in cardiovascular disease, risk factors, and their control in older women: British Women’s Heart and Health Study. *J. Epidemiol. Community Health* **57**, 134–140.

Marmot, M., and Brunner, E. (2005). Cohort Profile: the Whitehall II study. *Int. J. Epidemiol.* **34**, 251–256.

Montgomery, S.B., Sammeth, M., Gutierrez-Arcelus, M., Lach, R.P., Ingle, C., Nisbett, J., Guigo, R., and Dermitzakis, E.T. (2010). Transcriptome genetics using second generation sequencing in a Caucasian population. Nature 464, 773–777.

Robinson, W.P., et al. (1991). Three-locus systems impose additional constraints on pairwise disequilibria. *Genetics* **129**, 925–930.

Rodriguez, S., et al. (2014). Lipids, obesity and gallbladder disease in women: insights from genetic studies using the cardiovascular gene-centric 50K SNP array. EJHG, accepted.

The 1000 Genomes Project Consortium, et al. (2010). A map of human genome variation from population-scale sequencing. *Nature* **467**, 1061–1073.

Voight, B.F., et al. (2012). The metabochip, a custom genotyping array for genetic studies of metabolic, cardiovascular, and anthropometric traits. *PLoS Genet.* **8**, e1002793.

Walker, M., et al. (2004). The British Regional Heart Study 1975-2004. *Int. J. Epidemiol.* **33**, 1185–1192.

Marmot, M., and Brunner, E. (2005). Cohort Profile: the Whitehall II study. *Int. J. Epidemiol.* **34**, 251–256.

Wood AR et al. (2014). Defining the role of common variation in the genomic and biological architecture of adult human height. Nat Genet. 46 (11):1173-86.
